# miR‐1246 promotes osteosarcoma cell migration via NamiRNA‐enhancer network dependent on Argonaute 2

**DOI:** 10.1002/mco2.543

**Published:** 2024-04-07

**Authors:** Shuai Yang, Qingping Zou, Ying Liang, Dapeng Zhang, Lina Peng, Wei Li, Wenxuan Li, Mengxing Liu, Ying Tong, Lu Chen, Peng Xu, Zhicong Yang, Kaicheng Zhou, Jianru Xiao, Hailin Wang, Wenqiang Yu

**Affiliations:** ^1^ Shanghai Public Health Clinical Centre and Department of General Surgery Huashan Hospital Cancer Metastasis Institute and Laboratory of RNA Epigenetics Institutes of Biomedical Sciences Shanghai Medical College Fudan University Shanghai China; ^2^ State Key Laboratory of Environmental Chemistry and Ecotoxicology Research Centre for Eco‐Environmental Sciences Chinese Academy of Sciences Beijing China; ^3^ Department of Orthopaedic Oncology Changzheng Hospital Naval Medical University Shanghai China

**Keywords:** Argonaute 2, enhancer, metastasis, miR‐1246, nuclear activation miRNAs, osteosarcoma

## Abstract

High metastatic propensity of osteosarcoma leads to its therapeutic failure and poor prognosis. Although nuclear activation miRNAs (NamiRNAs) are reported to activate gene transcription via targeting enhancer and further promote tumor metastasis, it remains uncertain whether NamiRNAs regulate osteosarcoma metastasis and their exact mechanism. Here, we found that extracellular vesicles of the malignant osteosarcoma cells (143B) remarkably increased the migratory abilities of MNNG cells representing the benign osteosarcoma cells by two folds, which attributed to their high miR‐1246 levels. Specially, miR‐1246 located in nucleus could activate the migration gene expression (such as *MMP1*) to accelerate MNNG cell migration through elevating the enhancer activities via increasing H3K27ac enrichment. Instead, MMP1 expression was dramatically inhibited after Argonaute 2 (AGO2) knockdown. Notably, in vitro assays demonstrated that AGO2 recognized the hybrids of miR‐1246 and its enhancer DNA via PAZ domains to prevent their degradation from RNase H and these protective roles of AGO2 may favor the gene activation by miR‐1246 in vivo. Collectively, our findings suggest that miR‐1246 could facilitate osteosarcoma metastasis through interacting with enhancer to activate gene expression dependent on AGO2, highlighting the nuclear AGO2 as a guardian for NamiRNA‐targeted gene activation and the potential of miR‐1246 for osteosarcoma metastasis therapy.

## Graphical abstract

This study highlights that EVs‐derived miR‐1246 promotes osteosarcoma cell migration through activating MMP1 via NamiRNA‐enhancer network dependent on AGO2. AGO2 protects the miR‐1246/enhancer hybrids from degradation by RNase H to favor NamiRNA‐mediated gene activation. These findings suggest that miR‐1246 could facilitate osteosarcoma metastasis and serve as a potential target for treating osteosarcoma metastasis.

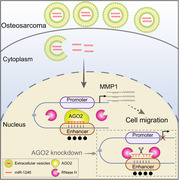

## 1 INTRODUCTION

Osteosarcoma is the most common original bone malignancy in pediatric ages and adolescents. It is reported that approximately 50% died patients couples with distant metastasis to the specific tissues and organs such as lungs and lymph nodes.^1^, ^2^ At present, clinical therapeutic options for osteosarcoma remain mainly involved in surgery, chemotherapy, and radiotherapy,^3^ but these clinical treatments do not considerably improve the prognosis of osteosarcoma patients due to its high metastatic propensity during the past 30 years.^1^ Thus, it is crucial for us to investigate the potential mechanisms underlying osteosarcoma metastasis and identify novel targets for clinical therapy.

MicroRNAs (miRNAs) are endogenously short noncoding RNAs that modulate gene expression.^4^, ^5^, ^6^ In recent years, some miRNAs associated with metastasis have been considered as potential therapeutic targets for osteosarcoma treatment. For instance, miR‐21 could significantly promote the invasion of osteosarcoma cells via indirectly increasing the expression of genes relevant to PI3K/AKT signaling pathway.^7^ Another report is that miR‐19a could directly decrease the *RhoB* expression and further result in osteosarcoma cell metastasis.^8^ Notably, miRNAs can be packaged in extracellular vesicles (EVs) to regulate osteosarcoma progression.^9^ Specifically, miR‐146a‐5p in osteosarcoma‐derived EVs could facilitate the distant osteosarcoma metastasis by suppressing the TRAF6 expression to prevent the maturation of osteoclast.^10^ Accordingly, investigation of the biological function of miRNAs in EVs and their gene regulatory patterns may provide alternative therapeutic strategies for osteosarcoma metastasis.

Recently, a series of miRNAs in nucleus were identified to have the potential of activating gene transcription as enhancer triggers,^5^, ^6^, ^11^, ^12^ named as nuclear activation miRNAs (NamiRNAs). One of the NamiRNAs, miR‐24‐1 activates gene transcription (such as *FBP1* and *FANCC*) by increasing the enrichment of histone H3 lysine 27 acetylation (H3K27ac) at enhancer loci.^5^ Interestingly, NamiRNAs could activate diverse gene transcription to regulate cancer progression. For example, NamiR‐26A1 reactivated VILL expression to suppress cell metastasis and proliferation in non‐small cell lung cancer.^13^ Moreover, loss of NamiRNA‐339 directly induced the downregulation of tumor suppressor genes including *YAP1*, *DUSP6*, and *GPER1*, and thus promoted the development of breast cancer.^6^ Of note, depletion of Argonaute 2 (AGO2) blocked these gene transcriptional activation, indicating that AGO2 is a key player in NamiRNA‐mediated gene activation. Remarkably, AGO2 can bind the RNADNA hybrids in cytoplasm,^14^ implying that AGO2 may directly interact with the RNA–DNA hybrids/loops to mediate NamiRNA‐targeted transcriptional activation in some way. Besides, AGO2 knockdown could significantly inhibit the migratory ability of osteosarcoma cells,^15^ indicating that AGO2 may regulate osteosarcoma metastasis. However, it remains uncertain whether EVs‐derived miRNAs participate in osteosarcoma metastasis as NamiRNAs and how AGO2 regulates NamiRNA‐targeted transcriptional activation in this procedure.

Here, we found that 143B cells possessed a more aggressive state than MNNG cells and the EVs separated from 143B cells significantly promoted MNNG cell migration. Then, miRNA microarray revealed that miR‐1246 was not only one of the top 10 miRNAs in EVs of 143B, but also the most upregulated miRNA compared with MNNG cells. Notably, miR‐1246 could promote different tumor metastasis such as lung cancer,^16^ esophageal squamous cell carcinoma,^17^ and oral squamous cell carcinoma.^18^ Thus, we chose EVs‐derived miR‐1246 as an example of the potent NamiRNAs to clarify how NamiRNAs modulate osteosarcoma metastasis and explore the roles of AGO2 during this process. Significantly, EVs‐derived miR‐1246 enhanced the migration of MNNG cells by upregulating migration genes. Furthermore, interaction between miR‐1246 and enhancer DNA loci in nucleus promoted gene transcription with the assistance of AGO2. Moreover, the binding of AGO2 to the miRNA/ssDNA hybrids protected their degradation from RNase H. Accordingly, AGO2 could act as a guardian to make sure the normal procedure of NamiRNA‐mediated transcriptional activation in nucleus by stabilizing the miRNA/ssDNA hybrids during osteosarcoma metastasis.

## 2 RESULTS

### 2.1 EVs mediate the transition of metastatic phenotype in osteosarcoma cells

**FIGURE 1:**
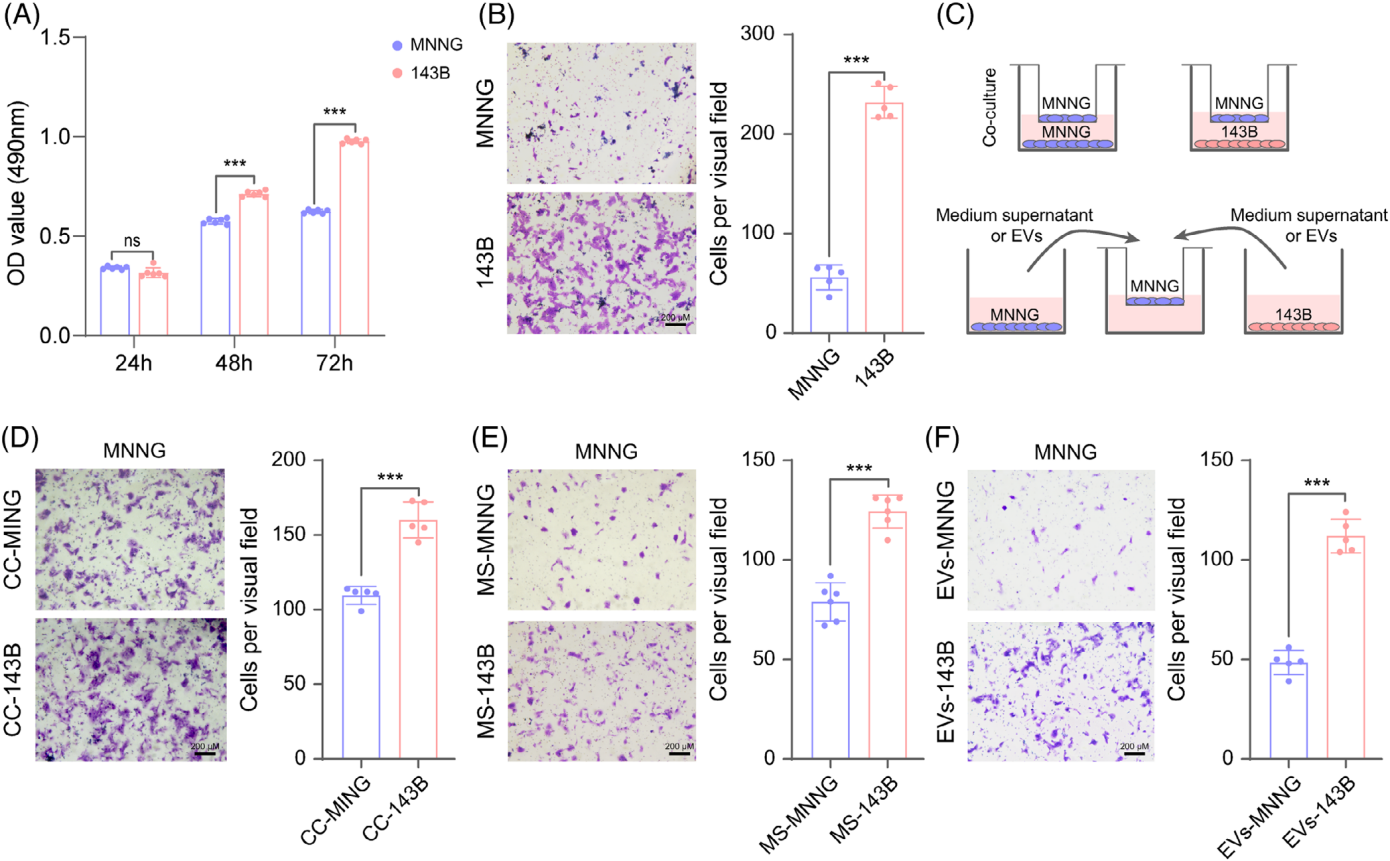
Extracellular vesicles facilitate the alteration in aggressive state of osteosarcoma cells. (A and B) Comparison of the cell proliferation (A) and migration (B) in 143B and MNNG cells. (C) Experimental scheme on the evaluation of MNNG cell migration under different conditions. Top: Coculture of MNNG cells with MNNG or 143B cells. Bottom: MNNG cells are cultured with medium supernatant (MS) or extracellular vesicles (EVs) from 143B cells. (D–F) Evaluation of the migratory ability in MNNG cells under different culture associated with 143B cells. Left: representative images of the MNNG cell migration under different culture conditions including noncontacting culture with MNNG cells (top) or 143B cells (bottom) in (D), incubation with the culture MS of MNNG cells (top) or 143B cells (bottom) in (E), and incubation with the extracellular vesicles derived from MNNG cells (top) or 143B cells (bottom) in (F). Scale bars, 200 µm. Right: quantification of the migrated MNNG cells under these conditions. Data are shown as mean ± Standard Deviation (SD). *p* alues are calculated using the Student's *t*‐test. *p < 0.05; **p < 0.01; ***p < 0.001; *ns*, not significant.

It is well known that tumor metastasis is a leading cause for the death of patients with osteosarcoma. Both of MNNG and 143B cells are osteosarcoma cell lines with the same genetic background, but they present different malignant degree.^19^, ^20^ As shown in Figures 1A and B, 143B cells possessed higher metastatic and proliferative abilities than MNNG cells, supporting that 143B cells are malignant osteosarcoma cells while MNNG cells are benign osteosarcoma cells. Here, we were curious whether the aggressive state can be transferred from 143B to MNNG cells. First, we cocultured MNNG cells with the equal numbers of 143B and MNNG cells through transwell assays (Figure 1C). Clearly, MNNG cells showed enhanced migratory ability after coculture with 143B cells than that with MNNG‐control cells (Figure 1D), indicating that the metastatic ability might be transferred between different osteosarcoma cell lines. Considering that the microenvironment is important for tumorigenesis and metastasis, we collected the medium supernatant (MS) of 143B or MNNG cells and added them to MNNG cells (Figure 1C). Similarly, MS of 143B cells significantly elevated the metastatic ability of MNNG cells compared with the MS of MNNG cells (Figure 1E). These results suggested that some unknown substances in the MS of 143B cells could promote MNNG cell migration.

EVs are cell‐derived membranous structures that are secreted from all cells for cell signal transduction and communication,^21^ and this pathway is relatively conserved from bacteria to plants and humans.^22^, ^23^ Intriguingly, EVs are reported to orchestrate multiple pathophysiological processes to facilitate tumor growth and metastasis.^24^ Accordingly, we extracted the EVs secreted by 143B cells in culture medium to assess their function on cell migration and proliferation in MNNG cells. Transmission electron microscopy showed that the EVs were successfully extracted from 143B cells, including exosomes and microvesicles (Figure S1A). To confirm the experimental stability of exosome extraction, we further extracted EVs from different cell lines (such as HEK293T, MNNG, and 143B cells) and detected the specific markers (such as CD9 and CD63) for EVs using western blot. As expected, both of CD9 and CD63 were enriched in EVs derived from different cells, but were almost undetectable in different cell protein extraction (Figure S1B). Then, EVs extracted from 143B cells were labeled with 3,3'‐dioctadecyloxacarbocyanine perchlorate (DIO) and added into the culture medium of HEK293T and MNNG cells. Clearly, EVs were rapidly absorbed by HEK293T and MNNG cells (Figure S1C). Subsequently, we evaluated the effects of EVs extracted from 143B and MNNG cells on MNNG cell migration (Figure 1C). Consistent with the effects of MS from 143B cells, EVs from 143B cells dramatically promoted MNNG cell migration (Figure 1F). However, the MS or EVs from 143B seemed to have no effect on cell proliferation in MNNG cells (Figures S1D and E). Collectively, all these above findings indicate that EVs are essential mediators to transit the metastatic phenotype from cell to cell.

### 2.2 EVs‐derived miR‐1246 from malignant osteosarcoma cells strengthens the cell migration of benign osteosarcoma

**FIGURE 2:**
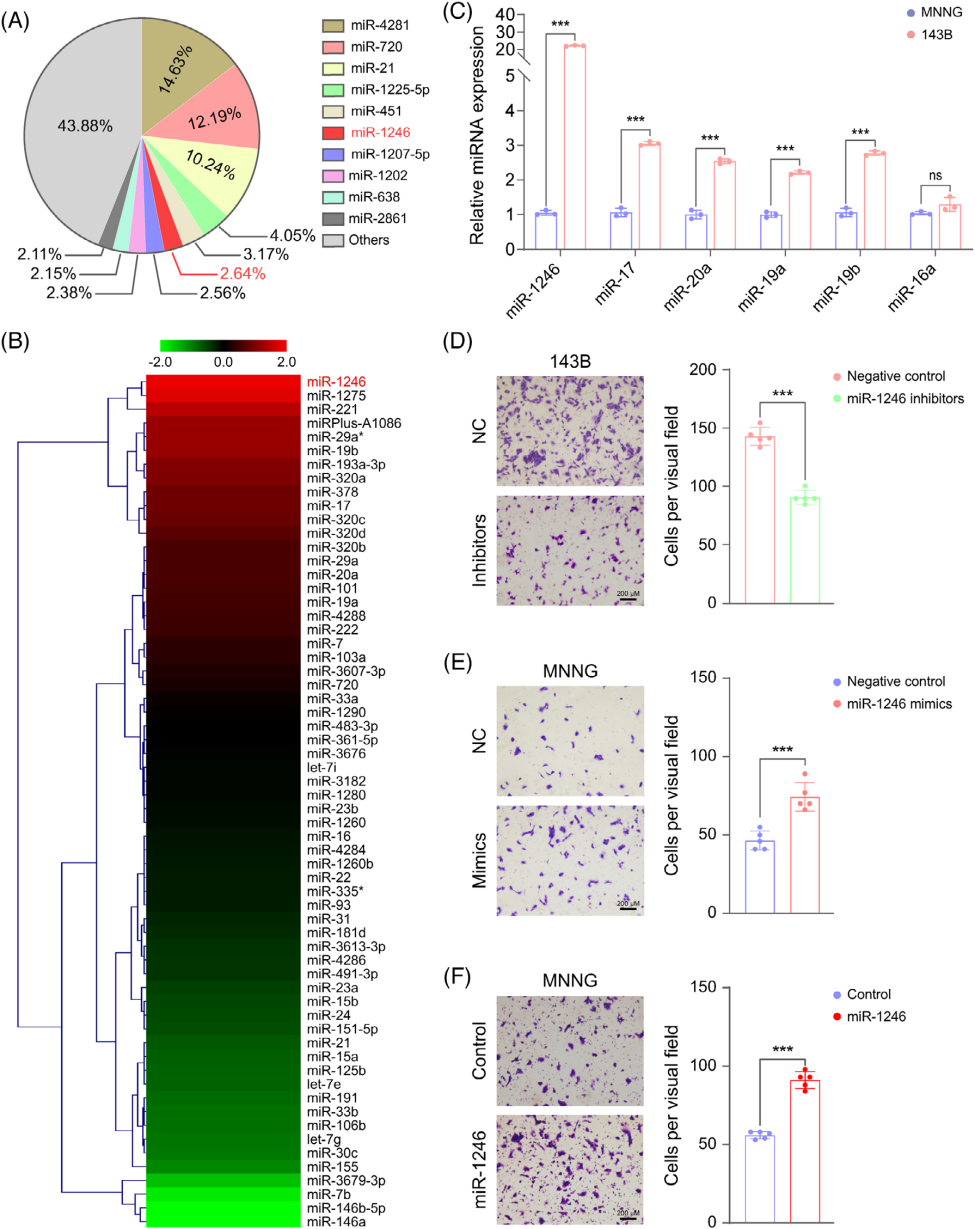
EVs‐derived miR‐1246 determines the migration potential of osteosarcoma cells. (A) Profile of miRNA enriched in extracellular vesicles of 143B cells. (B) Heatmap of the differentially enriched miRNAs between 143B and MNNG cells. (C) RT‐qPCR verifies the differential miRNAs between 143B and MNNG cells. (D–F) Detection of cell migration in 143B cells dealt with miR‐1246 inhibitors (D) or MNNG cells dealt with miR‐1246 mimics (E) or miR‐1246 overexpressed MNNG cells (F). Scale bars, 200 µm. The histograms showing the quantification of the migrated cell numbers of 143B or MNNG cells after these different treatments. Data in (C–F) are shown as mean ± SD. *p* values are calculated using the Student's *t*‐test. *p < 0.05; **p < 0.01; ***p < 0.001; *ns*, not significant.

In the past decade, growing evidence has emphasized that miRNAs packaged within EVs could regulate cell‐cell communication to affect cell function. For instance, highly abundant miRNAs (such as miR‐21‐5p) in EVs could promote the formation of premetastatic niche and tumor progression.^25^ To investigate whether miRNAs in 143B EVs could control the migration of MNNG cells, total RNA were extracted from 143B EVs, 143B, and MNNG cells, and detected by miRNA microarray. The top 10 miRNAs within 143B EVs were miR‐4281, miR‐720, miR‐21, miR‐1225‐5p, miR‐451, miR‐1246, miR‐1207‐5p, miR‐1202, miR‐638, and miR‐2861 (Figure 2A). Interestingly, miR‐1246 was one of the most significant changes in 143B cells in comparison with MNNG cells (Figure 2B), as well. In line with the miRNA microarray results, a series of tumor‐associated miRNAs (such as miR‐1246, miR‐17, miR‐20a, miR‐19a, and miR‐19b) were remarkably upregulated in 143B cells than MNNG cells while miR‐16a did not show significant difference (Figure 2C). Herein, we took miR‐1246 as an example to illustrate how miRNAs within EVs mediate the transition of metastatic phenotype from 143B to MNNG cells. At first, we transfected miR‐1246 inhibitors into 143B cells with a relatively high miR‐1246 expression. On the contrary, miR‐1246 mimics were transfected into MNNG cells with the low miR‐1246 expression. As shown in Figures S2A and B, RT‐qPCR showed that miR‐1246 inhibitors markedly decreased its expression in 143B cells while miR‐1246 mimics remarkably increased miR‐1246 expression in MNNG cells. Obviously, cell migration was decreased in 143B cells transfected with miR‐1246 inhibitors in comparison with negative control (NC) (Figure 2D). Conversely, miR‐1246 mimics significantly enhanced the migration of MNNG cells (Figure 2E). Likewise, the migratory ability of MNNG cells was also increased after transfection of the miR‐1246 expressed plasmid (Figure 2F). Meanwhile, miR‐1246 mimics could markedly promote the 143B cell migration whereas miR‐1246 inhibitors restrained cell migration of MNNG cells (Figures S2C and D), respectively. Subsequently, we treated MNNG cells with EVs from 143B and extracted the total RNA to evaluate the transition of miR‐1246. Clearly, EVs treatment significantly increased the expression of miR‐1246 (Figure S2E). Thus, these results demonstrate that EVs‐derived miR‐1246 may promote malignant metastasis of benign osteosarcoma through strengthening its cell migration.

### 2.3 miR‐1246 induces the migration of benign osteosarcoma cells through affecting migration gene expression

**FIGURE 3:**
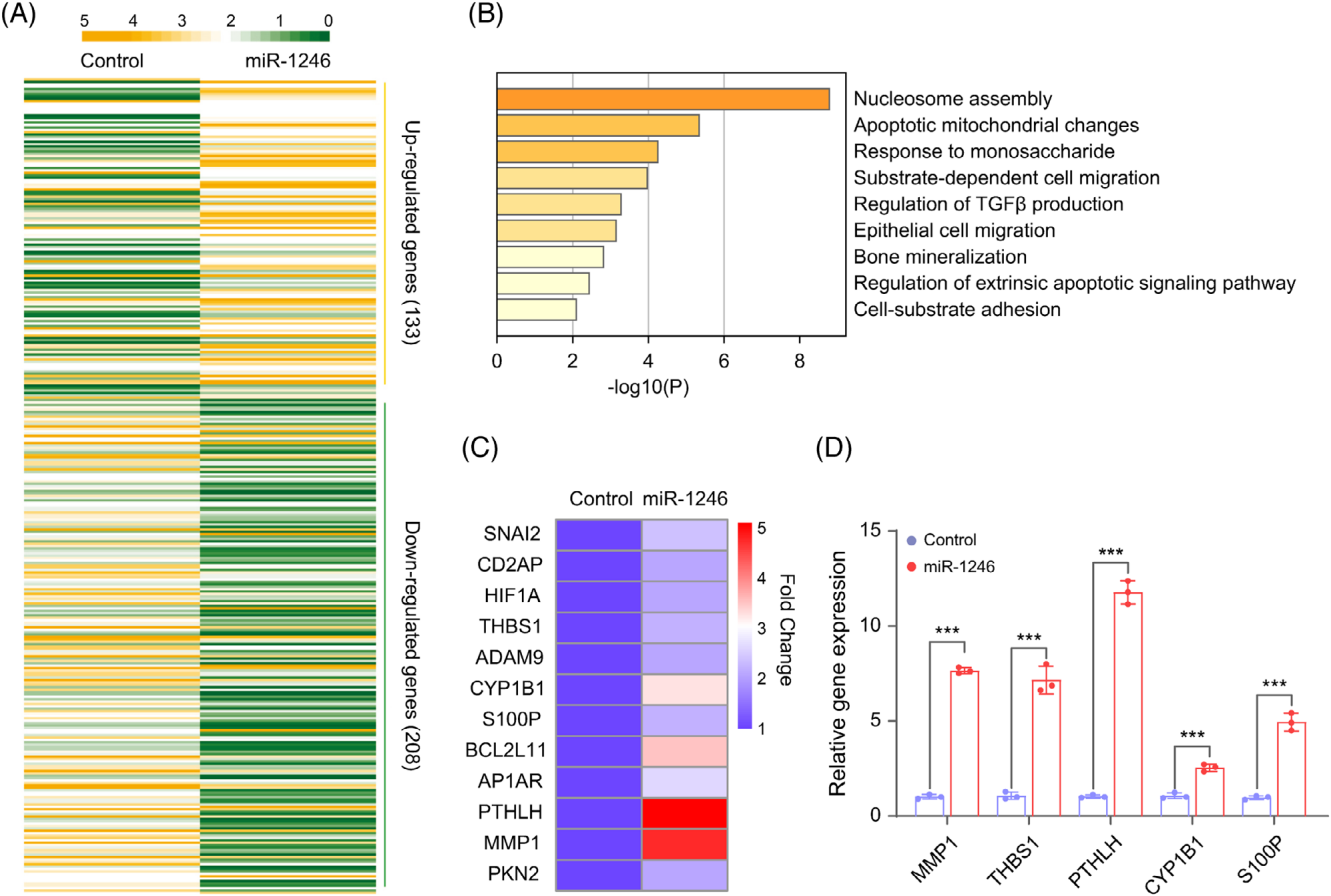
miR‐1246 promotes the osteosarcoma aggression via activating gene transcription in relation to cell migration. (A) Transcriptome analysis of the differential genes in miR‐1246‐overpressed MNNG cells. (B) Gene Ontology (GO) analysis on the upregulated genes after miR‐1246 overexpression in MNNG cells. (C) Differential expression genes for the GO terms associated with cell migration. (D) RT‐qPCR detects the mRNA expression of migration‐related genes in miR‐1246‐overpressed MNNG cells. Data are shown as mean ± SD. *p* values are calculated using the Student's *t*‐test. *p < 0.05; **p < 0.01; ***p < 0.001; *ns*, not significant.

Since discovery of the first miRNA lin‐4 in 1993,^26^ miRNAs are thought to be localized in the cytoplasm and regulate gene silencing at posttranscriptional level via targeting the 3′ UTRs of mRNA.^27^ Remarkably, recent investigation has indicated that NamiRNAs in nucleus could epigenetically activate gene transcription.^5^, ^6^, ^28^ To clarify the gene regulatory pattern of miR‐1246 in regulating cell migration in MNNG cells, we constructed the stable miR‐1246‐overexpressed MNNG cells. As shown in Figure S3A, miR‐1246 was significantly upregulated in this stable cell line when compared with the control cells. Then, we performed transcriptome sequencing to determine the differentially expressed genes (DEGs) caused by miR‐1246 overexpression. Heatmap exhibited that miR‐1246 could cause the upregulation of 133 genes (fold change ≥ 2) and the downregulation of 208 genes (fold change ≤ 0.5) in miR‐1246‐overexpressed MNNG cells (Figure 3A). These DEGs are presented in Table S1. Surprisingly, Gene Ontology (GO) analysis revealed that these upregulated genes were significantly associated with cell migration, such as “substrate‐dependent cell migration,” “epithelial cell migration,” and “cell‐substrate adhesion” (Figure 3B). These upregulated genes related to cell migration included *MMP1*, *THBS1*, *PTHLH*, *CYP1B1*, and *S100P* (Figure 3C), which were confirmed in MNNG cells with miR‐1246 overexpression using RT‐qPCR (Figure 3D). Instead, MMP1 expression was significantly reduced by miR‐1246 inhibitor treatment in 143B cells (Figure S3B), further supporting that miR‐1246 could specifically regulate MMP1 expression in osteosarcoma. As an important member of the matrix metalloproteinase family, MMP1 is closely related to cell metastasis.^29^ Notably, reduction of MMP1 could dramatically inhibit the metastatic ability of 143B cells.^30^ In addition, it has been revealed that miR‐1246 could enhance the metastasis of colorectal cancer by downregulating the expression of SPRED2.^31^ Consistently, the downregulated genes caused by miR‐1246 overexpression were also enriched in the terms with cell migration, such as “cell–matrix adhesion” and “extracellular matrix organization” (Figure S3C). For example, the expression of BCAM, IGFBP5, and PDPN were markedly decreased in miR‐1246‐overexpressed MNNG cells (Figure S3D). Meanwhile, BCAM suppressed the migration and invasion of hepatocarcinoma cells.^32^ IGFBP5 significantly reduced the cell migration and invasion of the trophoblast in preeclampsia^33^ while PDPN depletion directly promoted cell migration and invasiveness of thyroid carcinoma cells.^34^ Therefore, all these results support that miR‐1246 indeed regulates migration genes and further induce the benign osteosarcoma cell migration.

### 2.4 Nuclear miR‐1246 activates migration gene transcription through its interaction with enhancer dependent on AGO2

**FIGURE 4:**
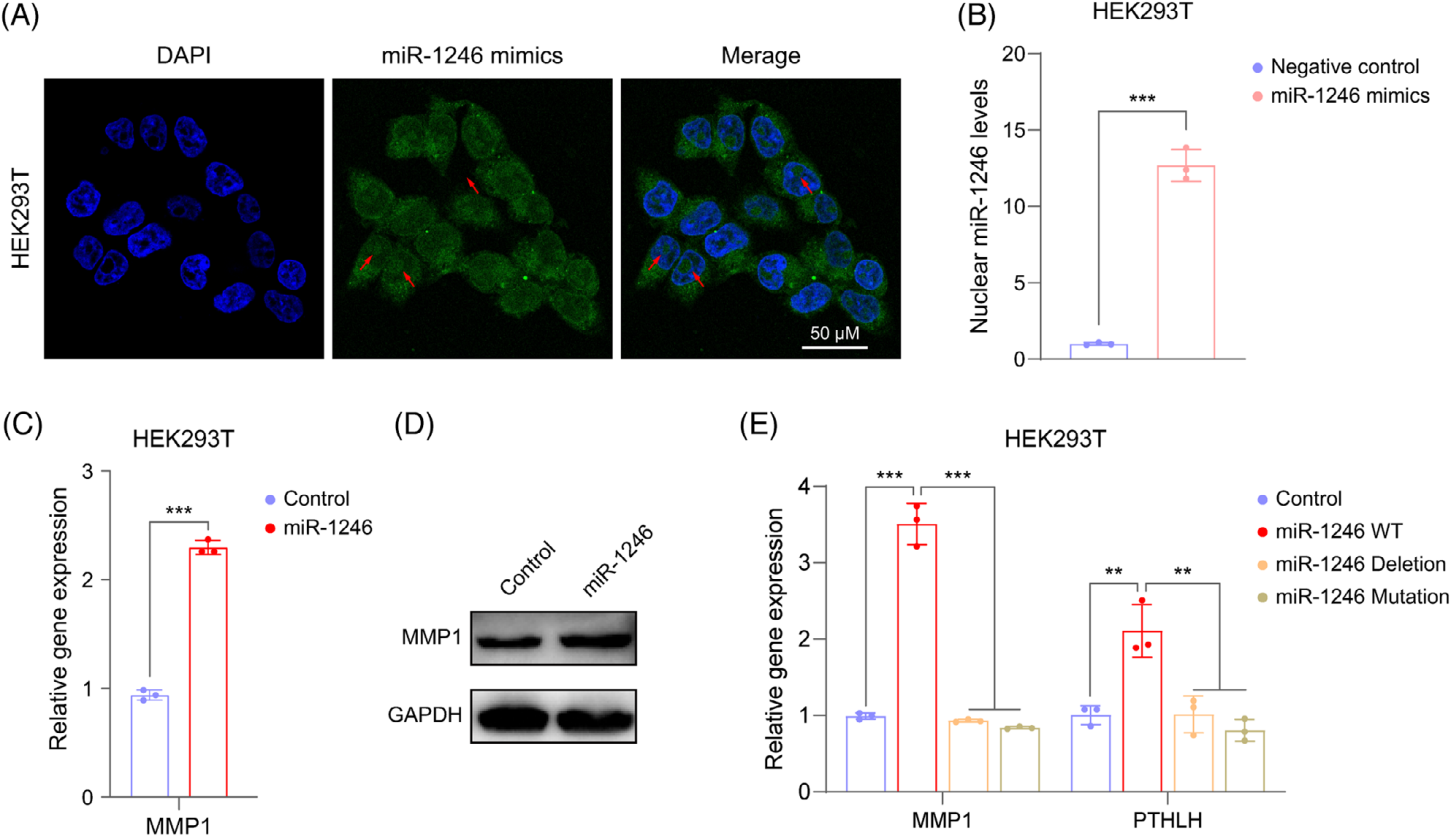
miR‐1246 in nucleus activates the transcription of migration genes in HEK293T cells. (A) Represented confocal images showing the location of miR‐1246 in cytoplasm and nucleus of HEK293T cells. The red arrows indicate the miR‐1246 mimics in the nucleus. Blue: DAPI; green: miR‐1246 mimics labeled with FAM. Scale bars, 50 µm. (B and C) RT‐qPCR detects the expression of nuclear miR‐1246 in miR‐1246 mimic transfected HEK293T cells (B) and MMP1 expression in miR‐1246‐overexpressed HEK293T cells (C). (D) Western blot verifies the expression of MMP1 in HEK293T cells after miR‐1246 overexpression. (E) RT‐qPCR assaying the MMP1 expression in HEK293T with the transfection of different plasmids. HEK293T cells are transfected with miR‐1246 expressed plasmid (WT), miR‐1246 expressed plasmids with deletion or mutation of miR‐1246, or empty plasmid (control). Data are shown as mean ± SD. *p* values are calculated using the Student's *t*‐test in (B and C) or one‐way ANOVA in (E). *p < 0.05; **p < 0.01; ***p < 0.001; *ns*, not significant.

Although NamiRNAs could activate gene transcriptional levels via targeting enhancer,^5^ the exact molecular mechanism remains not fully understood. One of the key points is whether miRNAs really exist in the cell nucleus. In this case, we bought miR‐1246 mimics labeled with FAM and transfected them into HEK293T and MNNG cells. Clearly, there was a colocalization of miR‐1246 and DAPI in HEK293T cells (Figure 4A) and MNNG cells (Figure S4A), proving that miR‐1246 could be located in nucleus. Besides, miR‐1246 was significantly elevated in the nucleus after transfection of miR‐1246 mimics (Figure 4B). Meanwhile, miR‐1246 mimics also enhanced MMP1 expression in HEK293T cells (Figure S4B). Consistent with this result, miR‐1246 overexpression using plasmid also enhanced the mRNA and protein levels of MMP1 in HEK293T cells (Figures 4C and D). To further confirm the specific activation of miR‐1246 on MMP1, we constructed different miR‐1246 expressed plasmids, including empty plasmid (control), miR‐1246 WT plasmid, miR‐1246 deletion plasmid, and miR‐1246 Mutation plasmid. Obviously, compared with control, miR‐1246 WT plasmids remarkedly promoted the expression of MMP1 in HEK293T cells, but the deletion or mutation of miR‐1246 did not cause the activation of MMP1 (Figure 4E). Thus, MMP1 was specifically activated by miR‐1246.

**FIGURE 5:**
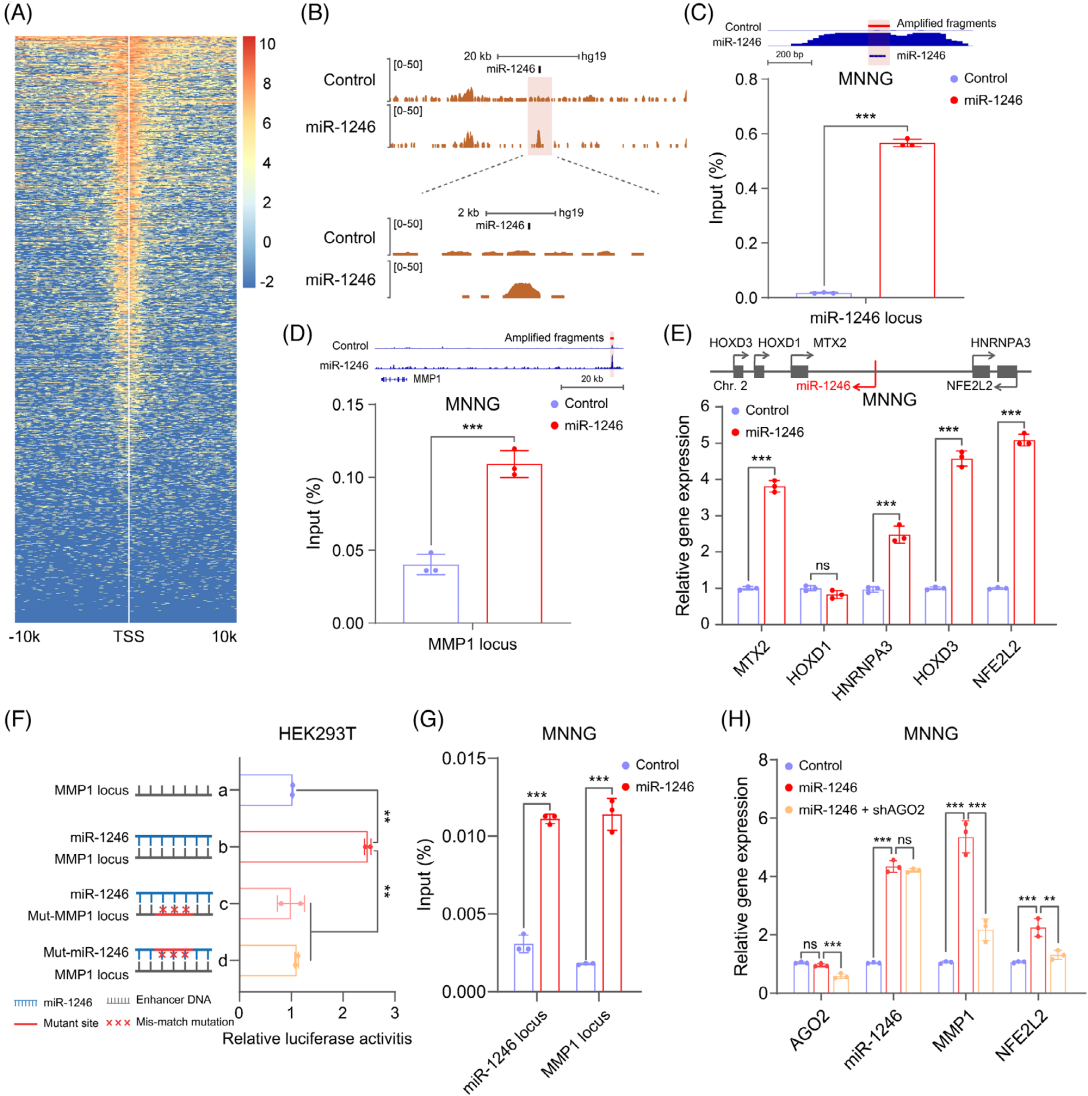
miR‐1246 activates gene transcription via targeting enhancer dependent on AGO2. (A) ChIP‐seq analysis for H3K27ac modification in miR‐1246‐overpressed MNNG cells. (B) Peak of H3K27ac modification at the genomic enhancer regions of miR‐1246 locus in miR‐1246‐overpressed MNNG cells. (C and D) ChIP‐qPCR confirms the enrichments of H3K27ac markers at the miR‐1246 (C) and MMP1 enhancer loci (D) in miR‐1246‐overpressed MNNG cells. IGV image showing the enrichment of H3K27ac at the corresponding regions. (E) RT‐qPCR determines the adjacent gene expression of miR‐1246 locus in miR‐1246‐overpressed MNNG cells. (F) Luciferase reporter assay assesses the enhancer activity of miR‐1246 and MMP1 loci in HEK293T cells using the indicated plasmids. (G) ChIP‐qPCR evaluates the AGO2 enrichment at the enhancer of miR‐1246 and MMP1 loci in miR‐1246‐overpressed MNNG cells. (H) RT‐qPCR determines the mRNA levels of genes activated by miR‐1246 in MNNG cells after knockdown of AGO2. Data are shown as mean ± SD in (C–H). *p* values are calculated using the Student's *t*‐test in (C–E and G) or one‐way ANOVA in (F and H). *p < 0.05; **p < 0.01; ***p < 0.001; *ns*, not significant.

As we all know, enhancers are *cis‐*regulatory sequences to control spatiotemporal and dynamic gene expression, which are not dependent on the orientation, and distance in relation to their target genes.^35^ To elucidate whether miR‐1246 can activate gene transcription via interaction with enhancer, chromatin immunoprecipitation (ChIP)‐seq was carried out using miR‐1246‐overexpressed MNNG cells with antibody against H3K27ac (Figure 5A), a well‐known marker for enhancer.^36^ It is clear that overexpression of miR‐1246 increased the H3K27ac enrichment at the miR‐1246 locus in MNNG cells (Figure 5B), which was verified by ChIP‐qPCR (Figure 5C). Noticeably, miR‐1246 remarkably elevated its precursor expression in cytoplasm and nucleus of MNNG cells (Figure S5A), indicating that miR‐1246 could activate itself expression. It is reported that P300 bromodomain‐dependent H3K27ac contributes to enhancer activities.^37^ We found that enrichment of P300 was observably increased at the enhancer regions of miR‐1246 loci (Figure S5B). Furthermore, ChIP‐seq and ChIP‐qPCR revealed that a potential enhancer site targeted by miR‐1246 was located at 70 kb upstream of MMP1 gene (Figure 5D), which was designated as the MMP1 enhancer locus. Subsequently, we found that some adjacent genes (such as *MTX2*, *HOXD3*, *HNRNPA3*, and *NFE2L2*) surrounding miR‐1246 locus were significantly activated by miR‐1246 in MNNG cells (Figure 5E). Moreover, we designed luciferase reporter assays to further investigate whether miR‐1246 could interact with its targeted enhancer loci in HEK293T cells. The fragment (760 bp) of miR‐1246 locus was cloned into pGL3‐Promoter vector (Enhancer WT) whereas its binding sites at the targeted enhancer regions (such as miR‐1246 and MMP1 loci) was mutated and inserted into pGL3‐Promoter vector (Enhancer Mutation). As expected, the luciferase activities were enhancive when we cotransfected miR‐1246 expression vector (miR‐1246 WT) with pGL3‐Enhancer‐WT (miR‐1246 or MMP1 loci) while mutation of miR‐1246 itself or its binding sites did not obviously affect the luciferase activities (Figures 5F and S5C), indicating that the enhancer integrity is key for the gene activation mediated by miR‐1246 and miR‐1246 could interact with the distal enhancers (such as MMP1 locus) in addition to its own enhancer. To further confirm the interaction between miR‐1246 and enhancer, we synthesized the miR‐1246 mimics with biotin‐modification at its 3′ terminal region and transfected them into MNNG cells for immunoprecipitation assays. After breaking DNA segments by sonication, we obtained the potential enhancer DNA fragments targeted by miR‐1246 using streptavidin‐conjugated beads. As shown in Figure S5D, the enhancer DNA was significantly enriched at MMP1 locus in MNNG cells transfected with biotin‐labeled miR‐1246 mimics, demonstrating that there existed interaction between miR‐1246 and enhancers in vivo. Therefore, miR‐1246 could interact with enhancers to promote gene transcription through increasing enhancer activities via inducing H3K27ac enrichment.

It is reported that AGO2 plays essential roles in miRNA‐mediated gene activation through interacting with enhancer.^5^, ^6^ Accordingly, we found that AGO2 was enriched at the enhancer loci of miR‐1246 and MMP1 in MNNG cells with miR‐1246 overexpression (Figure 5G), which was consistent with the results of the corresponding agarose gel electrophoresis (Figures S5E and F). Meanwhile, ChIP‐seq using antibodies against AGO2 or H3K27ac revealed that there was a simultaneous increased enrichment of AGO2 and H3K27ac at miR‐1246 enhancer locus (Figure S5G), implying that AGO2 might mediate the interaction between miR‐1246 and enhancer. To further evaluate whether AGO2 modulates miR‐1246 mediated gene activation, we constructed shRNA against AGO2 to inhibit its expression in miR‐1246 overexpressed MNNG cells. As shown in Figure 5H, knockdown of AGO2 could significantly inhibit the gene activation caused by miR‐1246 in MNNG cells, including *MMP1* and *NFE2L2*. Besides, it has been demonstrated that silencing AGO2 could significantly inhibit osteosarcoma cell migration,^15^ further supporting that AGO2 regulates osteosarcoma metastasis caused by miR‐1246 mediated gene activation.

Together, these results suggest that nuclear miR‐1246 can activate the adjacent and distant gene transcription through interaction with enhancer dependent on AGO2.

### 2.5 AGO2 recognizes the miR‐1246/ssDNA hybrids to block RNase H‐mediated degradation

Accumulated evidence has demonstrated that AGO2 located in nucleus can inhibit or activate gene expression through targeting the genomic regions in combination with small RNAs.^38^, ^39^ Our results indicated that AGO2 participated in NamiRNA‐mediated gene activation, which may work as a model to investigate the exact biological function of nuclear AGO2 in regulating gene activation. Structural studies have revealed that AGO2 is mainly comprised of PAZ (PIWI–ARGONAUTE–ZWILLE), MID (middle) and PIWI domains.^40^ In particular, the domains of PAZ and MID can anchor the small RNAs via specific binding pockets while PIWI domain can degrade the target RNAs complementary to these bound small RNAs.^41^ Notably, PIWI domain may recognize the single strand RNA–DNA hybrids due to its structure similar to RNase H. Thus, we hypothesized that AGO2 could recognize and bind miRNA–ssDNA hybrids/loops to form a functional complex of AGO2–miRNA–DNA.

**FIGURE 6:**
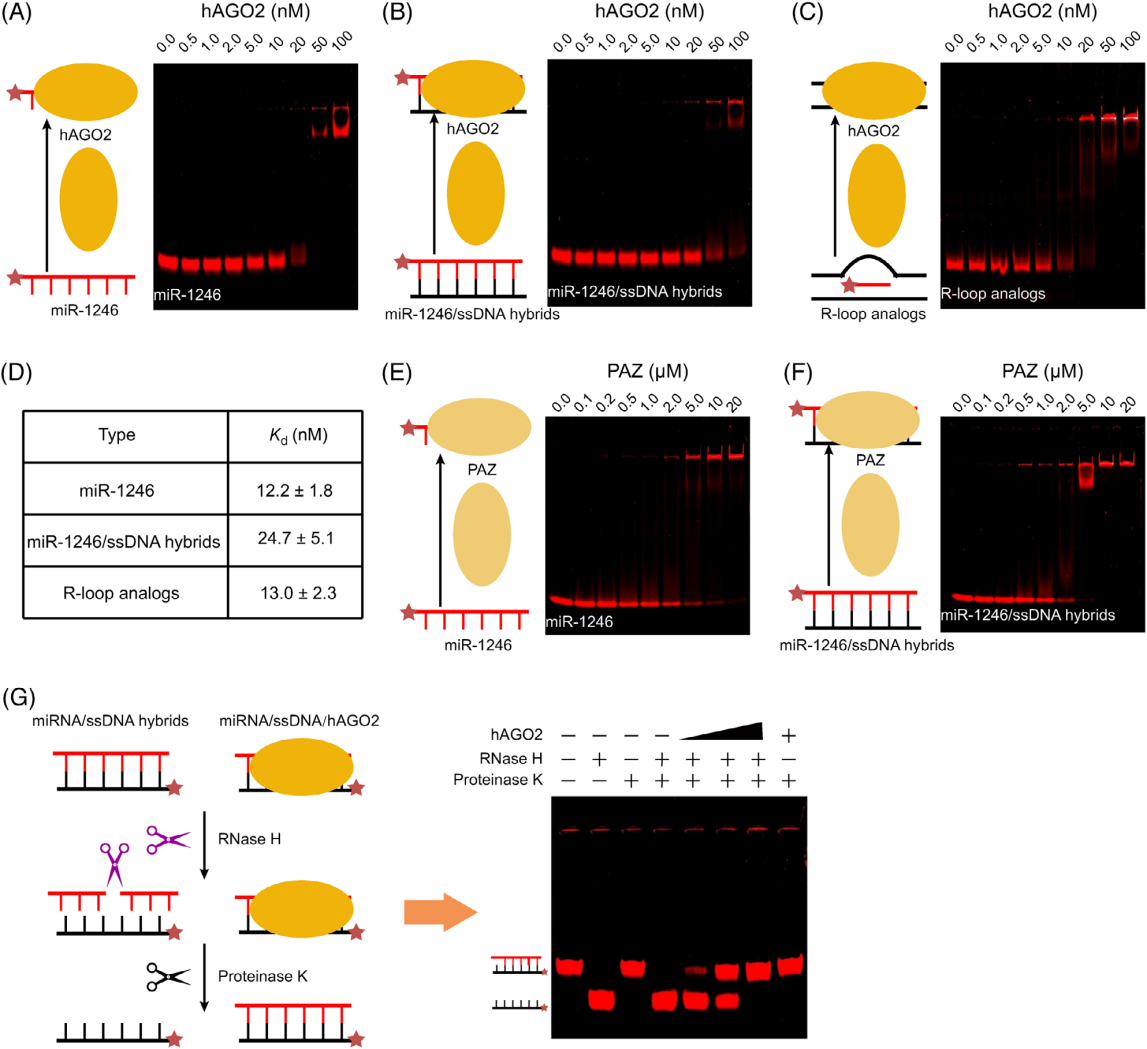
High affinity binding of AGO2 to miR‐1246/ssDNA hybrids blocks RNase H‐mediated degradation. (A–C) EMSA detects the affinity binding of AGO2 to miR‐1246 (A), miR‐1246/ssDNA hybrids (B), R loop analogs containing miR‐1246 (C). (D) The estimated dissociation constants (*K*_d_) for the binding of synthesized nucleic acid probes to hAGO2. (E and F) EMSA shows the affinity binding of PYZ domain of AGO2 to miR‐1246 (E), miR‐1246/ssDNA hybrids (F). (G) Left: schematic diagram of RNase H cleavage assay on the complex of AGO2 with miR‐1246/ssDNA hybrids. Right: EMSA evaluates the potential protective roles of AGO2 for the stability of miR‐1246/ssDNA hybrids against RNase H. In these EMSA experiments, miR‐1246 or ssDNA are labeled with CY5 to show their migration. miR1246/ssDNA hybrids are formed by annealing of miR‐1246 with its complementary ssDNA. R loop analogs are the miR‐1246/DNA loop mimics.

To validate our hypothesis, we designed a series of electrophoretic mobility shift assays (EMSA). First, we evaluated the binding of hAGO2 protein to miR‐1246 via labelling a CY5 fluorophore at its 5′ terminus. As shown in Figure 6A, free miR‐1246 was gradually decreased along with the increasing concentration of hAGO2. Then, we examined the binding activity of the purified recombinant hAGO2 protein to miR‐1246/ssDNA hybrids. Similarly, free miR‐1246/ssDNA hybrids were obviously reduced coupled with the increased hAGO2 concentration (Figure 6B), which is also suitable to the free R‐loop analogs (Figure 6C). Besides, the estimated dissociation constants (*K*_d_) for miR‐1246, miR‐1246/ssDNA hybrids, and R‐loop analogs were 12.2 ± 1.8, 24.7 ± 5.1, and 13.0 ± 2.3 nM, respectively (Figure 6D). Furthermore, we determined the binding of PAZ domain in AGO2 to miR‐1246 or miR‐1246/ssDNA hybrids in consideration of its binding ability on small RNAs. Expectedly, PAZ domain could bind to miR‐1246 or miR‐1246/ssDNA hybrids dependent on its concentration (Figures 6E and F). Noteworthily, the binding of PAZ domain showed stronger binding to miR‐1246/ssDNA hybrids than miR‐1246. These results supported that AGO2 can bind to miRNA/ssDNA hybrids and exert some biological function.

In fact, RNA/DNA hybrids abundantly exist in all cells of the living organisms during the synthesis processes of DNA and RNA,^42^ and RNase H could degrade the RNA in these hybrids independent on the sequences.^43^, ^44^ In this situation, we speculated that AGO2 may serve as a potential guardian to protect functionally the miRNA/ssDNA hybrids from RNase H cleavage during NamiRNA‐mediated gene activation. To confirm this assumption, we performed RNase H digestion assays to evaluate the stability of miR‐1246/ssDNA hybrids with or without AGO2 (Figure 6G). Surprisingly, miR‐1246 strand was well protected in miR‐1246/ssDNA hybrids from RNase H cleavage dependent on the dose of hAGO2 (Figure 6G). In contrast, the hybridized miR‐1246 strand was completely cleaved from the miR‐1246/ssDNA hybrid by RNase H in the absence of hAGO2 (Figure 6G). Therefore, all these findings in vitro hint that AGO2 determines the fate of miRNA–DNA (enhancer) hybrids in vivo through blocking their degradation from RNase H and thus regulates the NamiRNA‐mediated gene activation.

## 3 DISCUSSION

Although great progress has been made in osteosarcoma therapy, the prognosis of patients with osteosarcoma is still unoptimistic due to its high metastasis and heterogeneity.^45^, ^46^ Previous researches have highlighted that NamiRNAs could regulate the activation of oncogenes or antioncogenes during tumorigenesis.^6^, ^13^, ^47^ However, little is known about this NamiRNA‐mediated gene regulatory network during osteosarcoma metastasis. Here, we found that EVs secreted from malignant osteosarcoma cells could directly mediate the transition of the metastatic phenotype of benign osteosarcoma cells, which was partly due to the enrichment of miR‐1246 in EVs. Further experiments revealed that nuclear miR‐1246 accelerated MNNG cell migration through upregulating migration genes via targeting its enhancers dependent on the AGO2 protein, highlighting the therapeutic potential of miR‐1246 for osteosarcoma.

In this study, we revealed that EVs‐derived miRNAs including miR‐1246 could be important therapeutic targets for clinically blocking osteosarcoma metastasis. In recent years, EVs have attracted more attention for their important roles in different diseases (such as various cancer) and development.^24^, ^48^, ^49^ When it comes to osteosarcoma, proteomic analysis revealed that the secreted proteins in exosomes modulate osteosarcoma cell adhesion and migration.^50^ Additionally, transcriptome sequencing of EV RNAs pointed out that dramatic transcriptomic alterations appeared in metastatic and primary osteosarcoma.^51^ In line with these findings, we found that EVs from high metastatic osteosarcoma cell line 143B could accelerate MNNG cell migration and identified a series of abundant miRNAs packaged within these EVs related to its aggressive characteristics. For example, miR‐4281 and miR‐720 are also significantly upregulated in cutaneous malignant melanoma.^52^ Similarly, miR‐21 remarkably facilitates the metastasis and immune escape of osteosarcoma.^53^ Notably, we found, one of the abundant miRNAs in EVs, miR‐1246 was also the most increased miRNA in 143B cells compared with MNNG cells. Functional experiments demonstrated that block of miR‐1246 markedly inhibited 143B cell migration while miR‐1246 overexpression accelerated the metastatic ability of MNNG cells. In accordance with our results, miR‐1246 promotes cell migration and invasion of lung cancer.^16^ Besides, we found that the 133 upregulated genes (such as MMP1) caused by miR‐1246 in MNNG cells were enriched significantly in the GO terms of cell migration, resulting in the aggressive phenotype of MNNG cells. Specially, miR‐1246 could significantly activate *MMP1* expression in MNNG cells. In line with the biological function of miR‐1246 on osteosarcoma metastasis, knockdown of MMP1 in 143B cells inhibited the primary osteosarcoma metastasis in the lungs while MMP1 overexpression in nonmetastatic HOS cells facilitated the lung metastasis of osteosarcoma.^54^ Accordingly, miRNAs loaded within EVs could be pivotal regulators to orchestrate osteosarcoma metastasis and act as important candidate targets to develop small nucleotide drugs for osteosarcoma therapy.

Our results demonstrated that EVs‐derived miR‐1246 could activate migration genes to induce osteosarcoma metastasis via NamiRNA‐enhancer network. It is reported that EVs‐derived miRNAs could be transferred into cells from the other cells to regulate different biological procedures via endocytosis or the fusion with the plasma membrane.^53^, ^55^, ^56^ And most current studies concentrated on the negative regulatory roles of miRNAs in cytoplasm on genes during osteosarcoma development,^1^, ^2^, ^57^ but ignored the potential positive regulation of NamiRNAs for tumorigenesis.^6^, ^13^, ^47^ In our study, we showed that DIO‐labeled EVs from 143B cells existed in MNNG cells while the FAM‐labeled miR‐1246 was presented at nucleus of MNNG cells, indicating that EVs‐derived miR‐1246 could be transmitted into nucleus. And this nuclear location of miR‐1246 may be accomplished due to the disappearance of nuclear membranes during cell cycle.^58^ Interestingly, miR‐1246 could activate the enhancer activities by inducing the H3K27ac enrichment at its targeted enhancer regions including miR‐1246 and MMP1 loci in MNNG cells. Meanwhile, cotransfection miR‐1246 with its targeted enhancer loci plasmids could increase the luciferase activities while mutation of miR‐1246 or the corresponding enhancer regions significantly decreased the activities of luciferases, indicating that interaction between miR‐1246 and enhancer could activate gene transcription. In this situation, miR‐1246 promoted the expression of miR‐1246 precursors and MMP1, which is similar to the upregulation of *GPER1* activated by miR‐339 in breast cancer.^6^ Instead, miR‐1246 inhibitors could reduce the MMP1 expression, highlighting its specific regulation on MMP1 in osteosarcoma. Besides, it has been already demonstrated that MMP1 could facilitate osteosarcoma formation and its lung metastasis in mouse models in vivo,^54^ further supporting that the activation of MMP1 by nuclear miR‐1246 (NamiR‐1246) was vital for osteosarcoma metastasis. In fact, in addition to osteosarcoma, NamiRNA‐mediated gene activation is also involved in the other tumorigenesis such as breast cancer,^6^ non‐small cell lung cancer,^13^ and pancreatic cancer,^47^ highlighting that NamiRNA‐mediated gene activation is a general biological pattern and NamiRNAs could serve as important therapeutic targets for diverse tumors.

Importantly, we uncovered a protective role of AGO2 on the hybrids of miRNA‐enhancer in nucleus for NamiRNA‐mediated transcriptional activation. Previous studies have precisely characterized the specialized function of cytoplasmic Argonaute proteins in diverse small‐RNA guided gene repression.^40^, ^59^ However, recent research found that AGO2 participates in the NamiRNA‐mediated gene activation,^5^, ^6^, ^28^ highlighting the potential of nuclear AGO2 on gene upregulation. For instance, knockdown of AGO2 directly restrained the *GPER1* activation caused by miR‐339.^6^ Similarly, we found that AGO2 knockdown directly reduced the gene expression (such as *MMP1* and *NFE2L2*) activated by NamiR‐1246, which may be attributed to the interaction between nuclear AGO2 and enhancers.^60^ Consistently, AGO2 was enriched at the enhancers of miR‐1246 and MMP1 loci with the increase of H3K27ac markers at these corresponding regions after miR‐1246 overexpression in MNNG cells, indicating that AGO2 probably exerts key roles in NamiRNA‐mediated gene activation through chromatin remodeling. Furthermore, AGO2 could recognize the hybrids of NamiR‐1246 and the corresponding single stranded enhancer DNA in a dose‐dependent pattern and form a ternary complex. In line with this sight, AGO2 also recognized the hybrids of short SARS‐CoV‐2 RNA elements and identical human DNA,^28^ which may be due to the recognition of its PIWI domain on the DNA‐RNA hybrids.^40^ To our surprise, the binding of AGO2 to these hybrids could resist against their degradation by RNase H in vitro. In other words, AGO2 loading into the hybrids of NamiRNA and enhancer may stabilize the binding of NamiRNA to its targeted enhancer by preventing their cleavage from RNase H in vivo and further favor the NamiRNA‐mediated transcriptional activation. Moreover, miR‐1246 remarkably induced the synchronous enrichment of P300 and AGO2 at its targeted enhancer regions. It is notable that AGO2 could directly interact with P300 to promote lung cancer progression.^61^ Given that P300 is contributable to the enrichment of H3K27ac at enhancers,^37^ AGO2 may recruit P300 to activate enhancer activities by increasing the H3K27ac markers. Subsequently, interaction between enhancer and gene promoters mediated by transcriptional factors and Mediator complex could promote gene transcription.^62^ Therefore, the protective roles of AGO2 on the hybrids of NamiRNA and enhancer DNA could provide a relatively long time for the recruitment of transcriptional factors or activators during the NamiRNA‐mediated transcriptional activation.

In addition, there are still some limitations in this study. (1) Although different experiments have indicated that EVs‐derived miR‐1246 could be transmitted into nucleus, the exact mechanism on how EVs‐derived miRNAs are transported into nucleus remains an interesting scientific issue and deserves more investigation in the future. (2) It is a huge challenge to directly detect the complexes of nuclear AGO2 and miRNA/enhancer hybrids in vivo due to the lack of the corresponding biological technologies. Accordingly, developing methods for the detection of these ternary complexes in vivo will provide achievable approaches to explore the interaction in DNA–RNA–Protein complexes. (3) The specific function of AGO2 in the recruitment of transcriptional factors or activators during NamiRNA‐mediated gene activation needs further investigation in the future.

Together, EV‐derived miRNAs (such as miR‐1246) could promote osteosarcoma metastasis via NamiRNA‐enhancer network and blocking their biological function could be effective approaches for the treatment of metastatic osteosarcoma. And AGO2 could maintain the stability of NamiRNA/enhancer hybrids and further recruit some mediators to guarantee NamiRNA‐mediated transcriptional activation, which deepens our insights into the unexpected activating roles of nuclear AGO2 beyond the RISC‐mediated gene silencing.

## 4 MATERIALS AND METHODS

### 4.1 Cell lines

Human embryonic kidney 293T (HEK293T), human osteosarcoma cell lines 143B‐GFP cells, and MNNG/HOS (MNNG)‐RFP were severally cultured using DMEM medium (HyClone; SH30243.01) in combination with 10% fetal bovine serum (FBS) (Gibco; 10270‐106) and 1% penicillin–streptomycin (HyClone; SV30010) at humidified atmosphere under the condition of 37°C with 5% CO_2_.

### 4.2 EVs extraction

Fresh serum was centrifuged to remove its EVs for the preparation of cell culture medium. We collected the cell MS for EVs extraction in every 2–3 days for a total amount of 100 mL before cell passage and stored at 4°C. This collected medium was centrifuged for 20 min in 1500×g at 4°C to remove cell debris. Then, the supernatant was transferred to the ultracentrifugation tube and centrifuged continuously for 2 h at 4°C and 110,000×*g*. After removing the supernatant carefully, a thin white film could be seen on the tube wall and was resuspended with 200 µL precooled PBS to gain EVs.

### 4.3 Plasmid construction and transfection

We amplified the 399 bp fragment of pri‐miR‐1246 from HEK293T genomic DNA and then cloned it into pCDH‐CMV‐MCS‐EF1a‐copGFP lentiviral vector as miR‐1246 WT vector for lentivirus package with ClonExpress® II One Step Cloning Kit (Vazyme; C112). Then, we harvested lentiviruses from HEK293T cells and used them to infect MNNG or HEK293T cells to construct the stable cell lines. Inhibitors or mimics of miR‐1246 or NC were obtained from RiboBio (Guangzhou, China). And Hieff Trans™ liposome transfection reagent (YEASEN; 40802ES02) was used to transfect these plasmids and miRNA inhibitors or mimics into HEK293T, MNNG, 143B cells according to the manufacturer's description. The detailed primers for plasmid construction are provided in Table S2.

### 4.4 RNA isolation and RT‐qPCR

Collected cells were lysed by the TRIzol reagent (Invitrogen; 10296028) to extract total RNA. Total RNA was reversed into cDNA with the PrimeScript™ RT reagent Kit (Takara; RR047A). And we detected the expression of miR‐1246 and other genes (such as *MMP1*, *MTX2*, *HOXD1*, *HOXD3*, *HNRNPA3*, *NFE2L2*, and *AGO2*) using the SuperReal PreMix Plus (SYBR Green) regent (TIANGEN; FP205). Subsequently, U6 and GAPDH was respectively used as the internal control for the calculation of the relative miRNAs and mRNA expression through 2^−ΔΔCt^ method. The primers for RT‐qPCR assay are listed in Table S3.

### 4.5 Western blot

Collected cells were lysed by sodium dodecyl sulfate (SDS) lysis buffer containing protease inhibitors (Roche; 04693132001) to extract total proteins, which were used to performed western blot as previous description.^8^ The targeted blots were labeled with primary antibodies against CD63 (ABclonal; A5271), CD9 (ABclonal; A1703), GAPDH (ABclonal; AC002), and then incubated with Goat anti‐Mouse IgG (ABclonal; AS003) or Goat anti‐Rabbit IgG (ABclonal; AS014) conjugated with horseradish peroxidase. And we added the enhanced chemiluminescence substrate (Thermo Fisher Scientific; 32109) to detect the relevant hybridization signals.

### 4.6 Luciferase reporter assay

Fragments containing the putative enhancers of miR‐1246 and MMP1 locus and their mutation were amplified and then cloned into the pGL3‐Promoter vector, respectively. HEK293T cells were transfected with different plasmids to carry out luciferase reporter assay. Specially, the pGL3‐miR‐1246 (MMP1) locus‐WT or mutational enhancer plasmids were cotransfected with the miR‐1246‐WT or mutation expressed plasmids into HEK293T cells using Hieff Trans™ liposome transfection reagent (YEASEN; 40802ES02). After 48 h transfection, we evaluated the luciferase activities using the Dual Luciferase Reporter Assay Kit (Promega; E1960).

### 4.7 Cell functional assays

Transwell and MTT assays were respectively performed to evaluate the abilities of cell migration and proliferation as previously reported.^8^ For transwell assay, we resuspended osteosarcoma cells with serum‐free DMEM and seeded them into the upper transwell chamber (BD Biosciences) meanwhile the top compartments were supplemented with 20% FBS DMEM to induce cell migration. The 0.1% crystal violet (Sigma; C0775) solution was used to stain cells after 100% methanol fixation at 24 or 36 h. Then, we randomly selected optical fields to calculate the migratory cells under a microscope. As for MTT assay, 2 × 10^5^ cells were planted on 24‐well plates with 500 µL 10% FBS DMEM, and then assessed its proliferation ability using 3‐(4,5‐dimethylthiazol‐2‐yl)‐2,5‐diphenyltetrazolium bromide regents at 1, 2, and 3 cultured days.

### 4.8 Chromatin immunoprecipitation

After wash twice with 1 × PBS, cells were fixed with 1% formaldehyde (Sigma; F8775) for 10 min at room temperature and stopped with 0.125 M glycine solution for 10 min. Then, cells were collected and lysed in cell lysis buffer (10 mM HEPES pH 7.9, 0.5% NP‐40, 1.5 mM MgCl_2_, 10 mM KCl, and 1 × cocktail) for 15 min on the ice. Nucleus was extracted and dissolved in nuclear lysis buffer (50 mM Tris–HCl pH 8.1, 10 mM EDTA, 0.3% SDS, and 1× cocktail). After sonication, chromatin fragments were incubated with anti‐H3K27ac antibody (Abcam; ab177178) or anti‐AGO2 (Thermo Fisher Scientific; PA5‐117725) antibody and Protein A magnetic beads (Invitrogen; 10002D) overnight at 4°C. The immunoprecipitated DNA was purified with DNA Purification Kit (Qiagen; 28106). The concentration of ChIP‐derived DNA was detected by Qubit 2.0 and for next‐generation sequencing. Then, quantitative PCR was carried out to verify the ChIP‐seq data and normalized to input DNA (see Table S4 for primer sequences).

### 4.9 Probes preparation

The DNA and RNA oligonucleotides (see Table S5) used for interaction with AGO2 protein were bought from Shanghai Sangon Biotech Co. Ltd. All dsDNA, dsRNA, miRNA/ssDNA hybrid, and R‐loop structure were prepared as previous description.^28^ Briefly, the oligonucleotides were mixed with their complementary strands in the hybridization buffer and then annealed through gradient cooling. Then, we collected the target probes through separating the annealed mixtures on 10 or 16% nondenaturing polyacrylamide gel according to the reference (Molecular Cloning: A Laboratory Manual, Fourth Edition, by Green MR, Sambrook J, 2012 Cold Spring Harbor Laboratory Press, Cold Spring Harbor, New York, USA).^63^

### 4.10 Protein purification

Full‐length human argonaute 2 (hAGO2) and its PAZ‐domain (224‐350 aa) coding sequence was amplified and cloned into a home‐reconstructed pMAL‐C5X expression vector at the *Bam*H I and *Hin*d III restriction endonuclease sites for protein purification as previous description.^28^ In brief, the expression of hAGO2 or PAZ‐domain protein was induced by the addition of 0.2 mM isopropyl‐1‐thio‐β‐d‐galactopyranoside overnight at 16°C. Next, the cells were lysed in 100 mL lysis buffer containing EDTA‐free protease inhibitor and we loaded them onto the 5 mL MBPTrap HP column after centrifugation. Furthermore, the MBP‐hAGO2 recombinant protein or MBP‐PAZ‐domain were eluted with wash buffer and digested by thrombin to remove MBP‐tag. Finally, we further purified these proteins using the 1 mL HisTrap HP column.

### 4.11 Electrophoretic mobility shift assay

The hAGO2 recombinant protein was incubated with oligonucleotide probes (20 nM) in the fresh prepared binding buffer on the ice for 30 min as previous description.^28^ We used the 5% nondenaturing polyacrylamide gels to separate the unbound substrate probes from the protein‐substrate complexes in 0.5 × TBE electrophoresis buffer at 100 V for 50 min. After electrophoresis, we used Odyssey CLx dual‐color IR‐excited fluorescence imaging system (LI‐COR, Lincoln, NE) to detect the resolved oligonucleotide probes in these gels. The *K*_d_ was calculated by the nonlinear fitting.

### 4.12 RNase H digestion assay

RNase H was added to digest the miRNA/ssDNA hybrid mixtures with or without hAGO2 at 37°C for 30 min. Then, proteinase K was added to these mixtures to digest the hAGO2 and the RNase H at 55°C for 1 h before electrophoresis. The resulting digested products were resolved on 16% nondenaturing polyacrylamide gels for miRNA/ssDNA hybrids in 0.5 × TBE electrophoresis buffer at 100 V.

### 4.13 Statistical analysis

The statistic difference of data was analyzed using Student's *t* test between two groups or one‐way Analysis of Variance (ANOVA) in multiple groups using GraphPad Prism (Version 5.0; GraphPad Software, Inc.). The *p* < 0.05 was used as the criterion of statistical significance. *p < 0.05, **p < 0.01, and ***p < 0.001.

## AUTHOR CONTRIBUTIONS

Wenqiang Yu and Hailin Wang conceived and designed this project. Shuai Yang, Qingping Zou, Ying Liang, Dapeng Zhang, Lina Peng, Mengxing Liu, Ying Tong, Lu Chen, Peng Xu, Zhicong Yang, and Kaicheng Zhou performed the experiments, while Shuai Yang, Qingping Zou, Ying Liang, Dapeng Zhang, Jianru Xiao, and Peng Xu analyzed the experimental data. Wei Li and Wenxuan Li analyzed the high‐throughput sequencing data and uploaded these data to Gene Expression Omnibus (GEO). Shuai Yang, Qingping Zou, Dapeng Zhang, and Ying Liang wrote the draft. All of authors contributed to revising this manuscript and approved the final version.

## CONFLICT OF INTEREST STATEMENT

The authors declare no potential conflict of interest.

## ETHICS STATEMENT

Not applicable.

## Supporting information

Supporting Information

## Data Availability

The data of ChIP‐seq and RNA‐seq in the current research can be gained from the National Center for Biotechnology Information's Gene Expression Omnibus (GSE164322). All data that support the findings of this study are available in the main text or the supplementary materials.
