# Supplementary material for: miR‐1246 promotes osteosarcoma cell migration via NamiRNA‐enhancer network dependent on Argonaute 2
Source: MedComm (2020). 2024 Apr 7;5(4):e543. doi: 10.1002/mco2.543 (PMC10999177; doi:10.1002/mco2.543)
Supplement: Supplementary file 1 — Supporting Information [file MCO2-5-e543-s001.docx]

Supporting Information for

**miR-1246 promotes osteosarcoma cell migration via NamiRNA-enhancer network dependent on Argonaute 2**

Shuai Yang^1,#^, Qingping Zou^1,#^, Ying Liang^1,#^, Dapeng Zhang^2,#^, Lina Peng^1^, Wei Li^1^, Wenxuan Li^1^, Mengxing Liu^1^, Ying Tong^1^, Lu Chen^1^, Peng Xu^1^, Zhicong Yang^1^, Kaicheng Zhou^1^, Jianru Xiao^3^, Hailin Wang^2,^*, Wenqiang Yu^1,^*

^1^Shanghai Public Health Clinical Centre and Department of General Surgery, Huashan Hospital, Cancer Metastasis Institute and Laboratory of RNA Epigenetics, Institutes of Biomedical Sciences, Shanghai Medical College, Fudan University, Shanghai, China

^2^State Key Laboratory of Environmental Chemistry and Ecotoxicology, Research Centre for Eco-Environmental Sciences, Chinese Academy of Sciences, Beijing, China

^3^Department of Orthopaedic Oncology, Changzheng Hospital, Naval Medical University, Shanghai, China

^#^These authors contributed equally: Shuai Yang, Qingping Zou, Ying Liang, Dapeng Zhang.

*Correspondence: hlwang@rcees.ac.cn (H.W.), wenqiangyu@fudan.edu.cn (W.Y.)

**This file includes:**

Figures S1 to S5

Tables S1 to S5


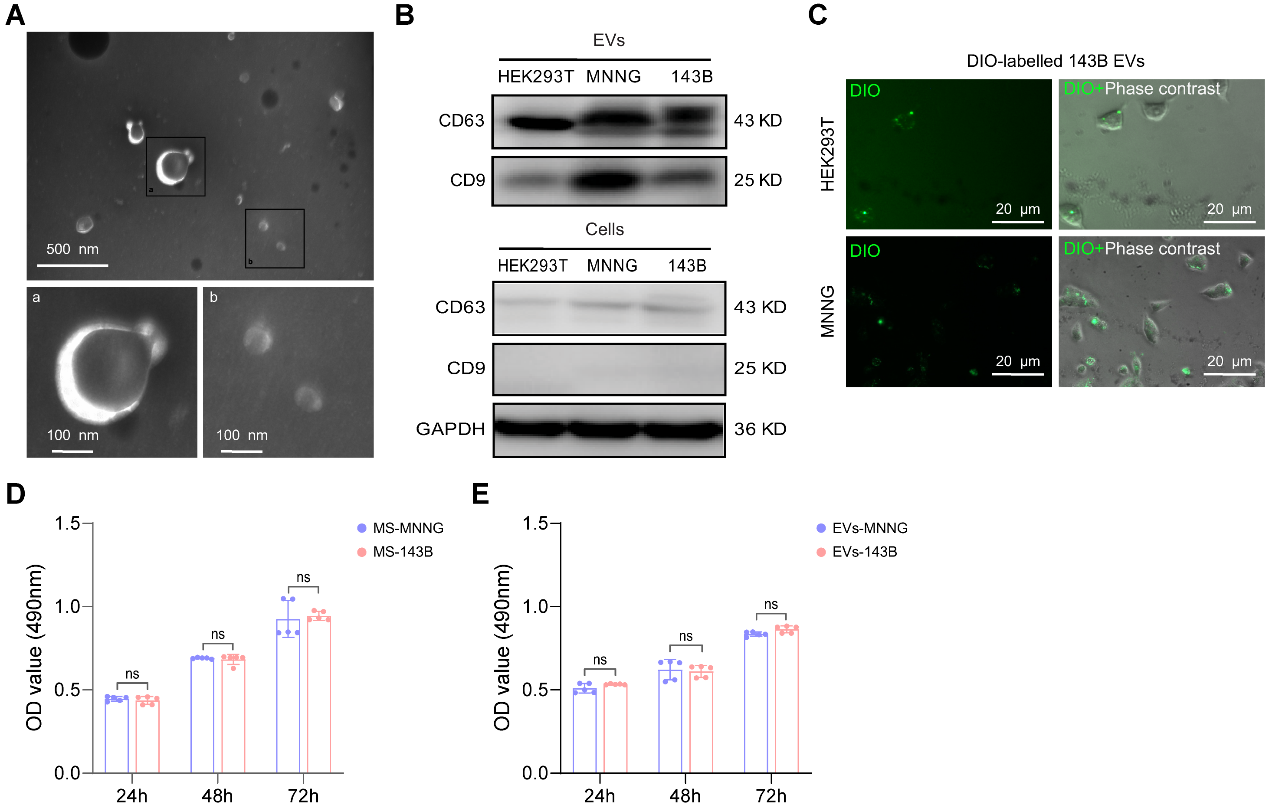


**FIGURE S1** The effects of extracellular vesicles on the cell proliferation of MNNG cells. (A) Transmission electron microscopy presents extracellular vesicles such as exosomes (white a) and microvesicles (white b) in 143B cells. Scale bars, 500 nm and 100 nm (enlargements). (B) Western blot determines the CD63 and CD9 markers in the extracellular vesicles (EVs) from HEK293T, MNNG, and 143B cells. (C) Fluorescence microscope observes the ingestion of DIO-labeled (green) EVs from 143B by HEK293T (Upper panel) and MNNG cells (Lower panel) after incubating for 24 h. Scale bars, 20 μm. (D-E) Evaluation of the cell proliferation in MNNG cells after treatment with culture medium supernatant (MS) (D) or EVs (E) from 143B cells. Data are shown as Mean ± SD. *P*-values are calculated using the Student’s *t* test. *, *P* < 0.05; **, *P* < 0.01; ***, *P* < 0.001; *ns*, not significant.


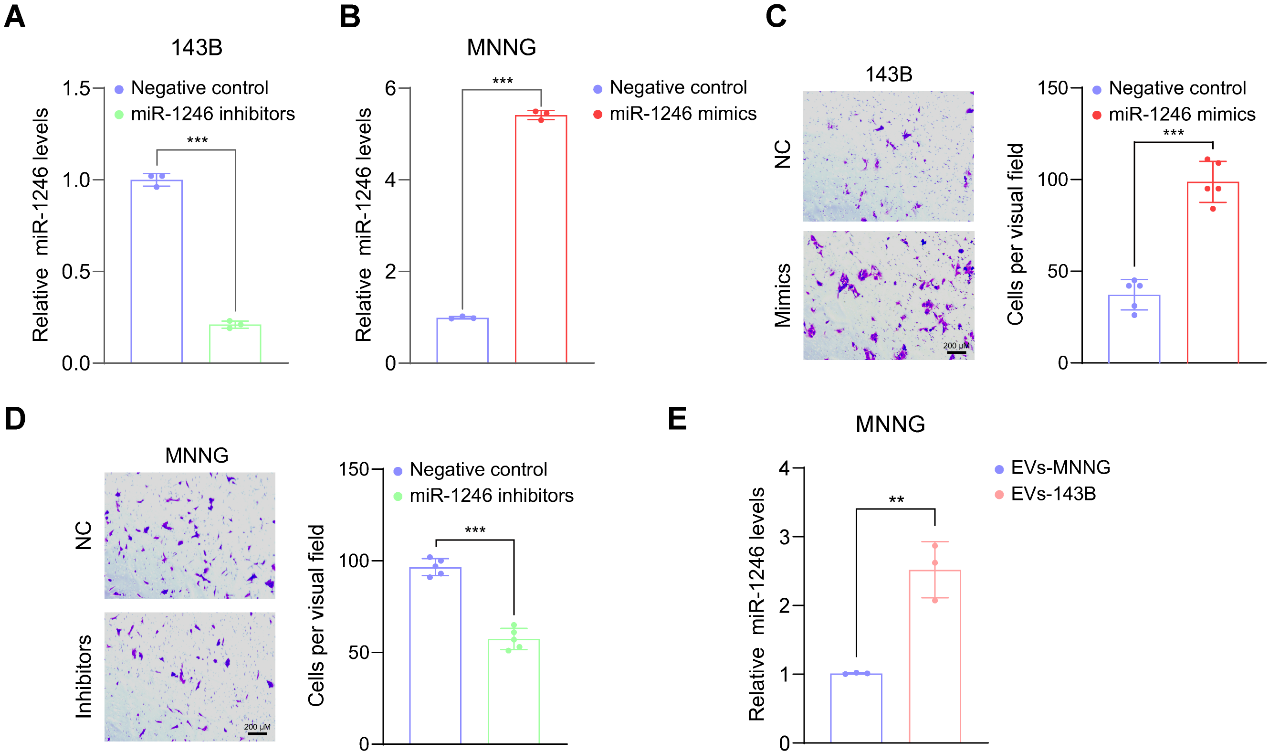


**FIGURE S2** Evaluation on the function of miR-1246 in osteosarcoma cell lines. (A-B) RT-qPCR detects the efficiency of miR-1246 mimics or inhibitors in 143B (A) and MNNG cells (B). (C-D) Transwell assays detect the effect of miR-1246 on cell migration in 143B (C) and MNNG (D) cells. (E) RT-qPCR determinates the miR-1246 expression in MNNG cells after EVs treatment. All data are shown as Mean ± SD. *P*-values are calculated using Student’s *t* test. *, *P* < 0.05; **, *P* < 0.01; ***, *P* < 0.001; ns, not significant.


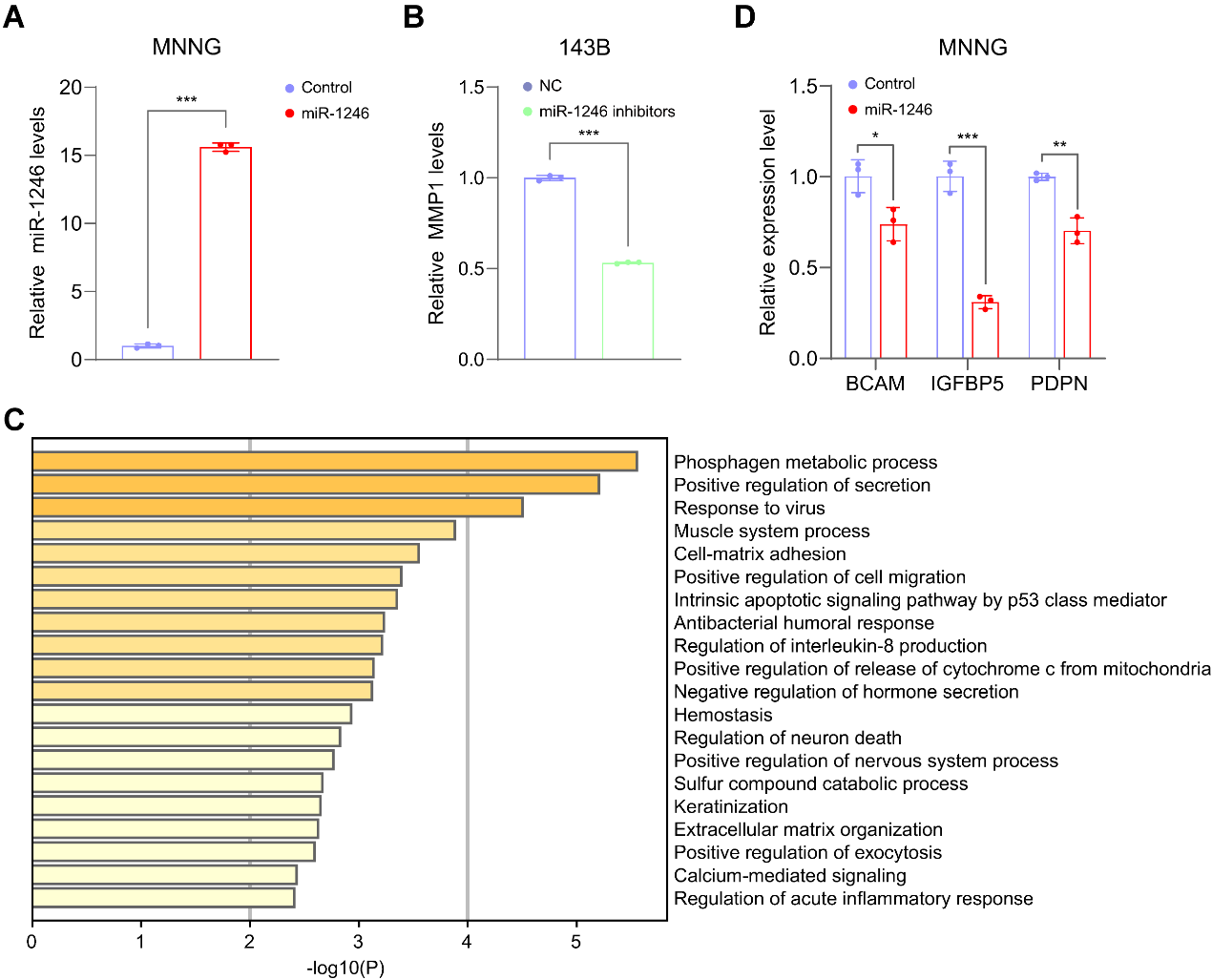


**FIGURE S3** Confirmation of the expression of miR-1246 and its downregulation genes in different cells. (A-B) Assessment of the miR-1246 levels in miR-1246-overexpressed MNNG (A) or 143B cells (B) after miR-1246 inhibitor transfection. (C) Gene Ontology (GO) analysis on the downregulated genes after miR-1246 overexpression in MNNG cells. (D) RT-qPCR verifies the mRNA levels of migration-related genes in miR-1246-overpressed MNNG cells. Data are shown as Mean ± SD in (A, B, and D). *P*-values are calculated using the Student’s *t* test. *, *P* < 0.05; **, *P* < 0.01; ***, *P* < 0.001; *ns*, not significant.


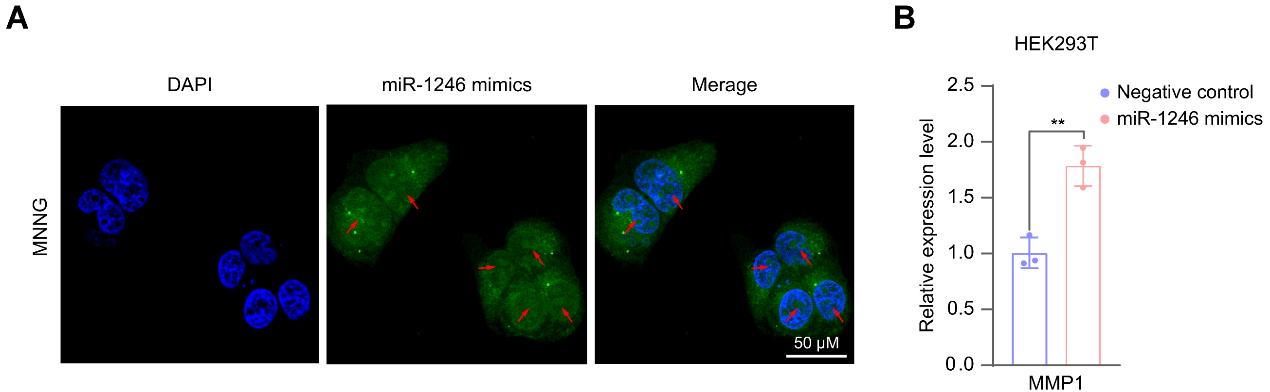


**FIGURE S4** The location of nuclear miR-1246 and its effect on MMP1 expression. (A) Represented confocal images showing the location of miR-1246 in cytoplasm and nucleus of MNNG cells. The Red arrows indicate the miR-1246 mimics in the nucleus. Blue: DAPI; Green: miR-1246 mimics labelled with FAM. Scale bars, 50 μm. (B) RT-qPCR detects the MMP1 expression in miR-1246 mimics transfected HEK293T cells. Data are shown as Mean ± SD in (B). *P*-values are calculated using the Student’s *t* test. *, *P* < 0.05; **, *P* < 0.01; ***, *P* < 0.001; *ns*, not significant.


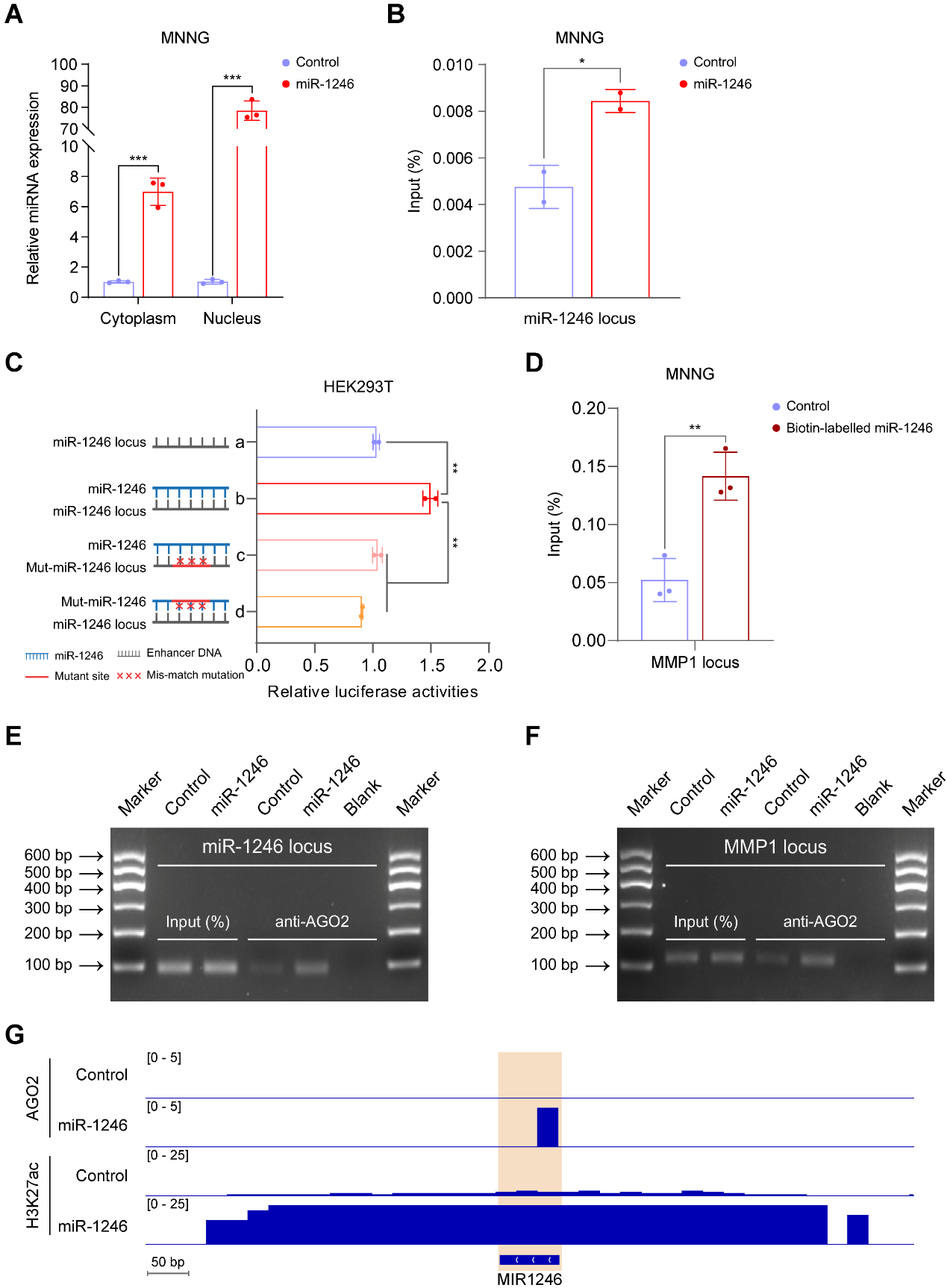


**FIGURE S5** Exploration of the interaction among miR-1246, host enhancer and AGO2. (A) RT-qPCR determining the expression of miR-1246 precursors in the cytoplasm and nucleus of miR-1246-overexpressed MNNG cells. (B) ChIP-qPCR assay detects the enrichment of P300 in miR-1246 locus in MNNG-miR-1246 cells. (C) Luciferase reporter assay assesses the enhancer activity of miR-1246 locus in HEK293T cells using the indicated plasmids. (D) qPCR detects the enrichment of enhancer DNA at MMP1 locus in biotin-labelled miR-1246 transfected MNNG cells. (E-F) Agarose gel electrophoresis confirms the enrichment of AGO2 at the enhancer sites of miR-1246 (E) and MMP1 (F) loci in miR-1246 overexpressed MNNG cells. DNA derived from ChIP assay is used as the templates in PCR while there are no templates in the Blank group. (G) IGV images showing the enrichment of AGO2 and H3K27ac enrichment at the miR-1246 locus in miR-1246-overexpressed MNNG cells. Data are shown as Mean ± SD in (A-D). *P*-values are calculated using the Student’s *t* test in (A, B, D) or one-way ANOVA in (C). *, *P* < 0.05; **, *P* < 0.01; ***, *P* < 0.001; *ns*, not significant.

**Table S1** Differentially expressed genes in MNNG cells with miR-1246 overexpression.

| Gene | Fold Change | Gene | Fold Change | Gene | Fold Change |
| --- | --- | --- | --- | --- | --- |
| COX7B | 66.69 | ADAM9 | 2.07 | TRAPPC9 | 0.46 |
| MIR7851 | 29.41 | TMEM200A | 2.06 | VEGFB | 0.46 |
| PGK1 | 9.60 | IFIT2 | 2.05 | IRF7 | 0.46 |
| MIR4532 | 6.92 | ZFAND1 | 2.05 | ARRB1 | 0.46 |
| SNORA80A | 6.21 | HIST1H2BG | 2.04 | SAPCD1 | 0.46 |
| MAGT1 | 5.80 | SNHG4 | 2.04 | NPIPB5 | 0.46 |
| SH3BGRL | 5.17 | MAD2L1 | 2.04 | TNFRSF25 | 0.45 |
| PTHLH | 5.10 | SNORD58C | 2.03 | THY1 | 0.45 |
| SNORA12 | 5.08 | BCDIN3D | 2.03 | IDUA | 0.45 |
| MMP1 | 4.74 | ZCCHC10 | 2.03 | SPAG4 | 0.45 |
| MIR3141 | 4.64 | UCHL5 | 2.02 | SULT1A3 | 0.45 |
| SNORD29 | 4.63 | CSGALNACT2 | 2.02 | B3GNT9 | 0.45 |
| MIR4792 | 4.61 | ITGA2 | 2.01 | CA11 | 0.45 |
| MIR4634 | 4.48 | GTF2H2B | 2.01 | FADS2 | 0.45 |
| MIR1234 | 4.34 | LOC100270746 | 2.01 | TMEM81 | 0.45 |
| HIST1H3B | 4.06 | HIST1H2BM | 2.01 | PTGES | 0.45 |
| SNORD15B | 4.05 | RHOT1 | 2.01 | ATP2A3 | 0.45 |
| MIR663A | 3.94 | SLC35F5 | 2.01 | LGALS3BP | 0.45 |
| LINC00273 | 3.83 | E2F5 | 2.00 | NPIPB3 | 0.45 |
| SNORD22 | 3.72 | TAPBP | 0.50 | PTP4A3 | 0.45 |
| ARSI | 3.65 | MYRF | 0.50 | OCEL1 | 0.45 |
| MIR3648 | 3.63 | HSF4 | 0.50 | SAP25 | 0.45 |
| HNRNPA1P10 | 3.60 | C2orf82 | 0.50 | PIANP | 0.44 |
| HIST2H2AB | 3.58 | CSF2 | 0.50 | LOC613037 | 0.44 |
| BCL2L11 | 3.53 | PKI55 | 0.50 | RHPN1 | 0.44 |
| MIR3661 | 3.48 | SNORA11D | 0.50 | CRIP2 | 0.44 |
| VAMP7 | 3.42 | ARHGAP33 | 0.50 | LOC643387 | 0.44 |
| CYP1B1 | 3.29 | EHD2 | 0.50 | FXYD6 | 0.44 |
| HIST1H4B | 3.28 | OPLAH | 0.50 | LINC01002 | 0.44 |
| HIST1H2AG | 3.25 | ASB16-AS1 | 0.50 | ADAMTSL4 | 0.44 |
| NKX2-3 | 3.15 | RBPMS2 | 0.50 | ANKS1B | 0.44 |
| HIST1H1B | 3.13 | GAA | 0.50 | TRPM4 | 0.44 |
| BMP8B | 3.10 | CNPY4 | 0.50 | LOC388849 | 0.44 |
| SNORA27 | 3.10 | COL18A1 | 0.49 | ANG | 0.44 |
| RPL21 | 3.08 | APOBEC3F | 0.49 | TP73 | 0.43 |
| ANXA10 | 3.05 | UPK3BL | 0.49 | TCN2 | 0.43 |
| LOC284801 | 3.03 | EEF1A2 | 0.49 | TMEM59L | 0.43 |
| HIST1H2AL | 3.01 | LRRC23 | 0.49 | C2orf81 | 0.43 |
| MIR663B | 2.97 | ANKZF1 | 0.49 | ZNF385A | 0.43 |
| MIR5047 | 2.85 | ZP3 | 0.49 | ITGB2 | 0.43 |
| DDX60 | 2.84 | VWA1 | 0.49 | CA9 | 0.43 |
| MAFA | 2.82 | CCDC159 | 0.49 | SAMD10 | 0.43 |
| RMRP | 2.81 | E2F2 | 0.49 | TUBB4A | 0.42 |
| ANKRD30BL | 2.78 | CITED4 | 0.49 | C16orf86 | 0.42 |
| CHAC1 | 2.77 | UCP2 | 0.49 | BCL3 | 0.42 |
| HNRNPUL2-BSCL2 | 2.77 | LOC729218 | 0.49 | CSAD | 0.42 |
| DKK1 | 2.75 | GNB3 | 0.49 | LINC00565 | 0.42 |
| LOC101928075 | 2.72 | MIR497HG | 0.49 | LOC100862671 | 0.42 |
| TEX101 | 2.66 | EBF4 | 0.49 | PCOLCE | 0.42 |
| PMAIP1 | 2.63 | TENC1 | 0.49 | CKMT1B | 0.42 |
| AP1AR | 2.62 | PLA2G6 | 0.49 | LOC100133331 | 0.42 |
| RNF138 | 2.60 | HSPB1 | 0.49 | SSC5D | 0.42 |
| IFI44 | 2.55 | ITGA7 | 0.49 | CFP | 0.42 |
| CDK17 | 2.54 | CKB | 0.49 | PGF | 0.41 |
| TSPAN7 | 2.52 | PYCARD | 0.49 | SH2B2 | 0.41 |
| SMARCA5-AS1 | 2.51 | LINC01001 | 0.49 | IGFBP5 | 0.41 |
| HIST2H3A | 2.51 | CCDC24 | 0.49 | TFEB | 0.41 |
| LYPLA1 | 2.48 | NRM | 0.49 | C19orf54 | 0.41 |
| ZFHX3 | 2.47 | SRRM3 | 0.49 | ZHX1-C8orf76 | 0.41 |
| HIST2H3C | 2.47 | DHRS4L1 | 0.49 | C6orf52 | 0.41 |
| SNORA71A | 2.45 | HOOK2 | 0.49 | MIR210HG | 0.41 |
| HIST1H3C | 2.41 | PARP6 | 0.48 | SEMA6B | 0.41 |
| SCARNA2 | 2.40 | LYRM9 | 0.48 | LTB4R | 0.40 |
| SNORD51 | 2.40 | IFITM3 | 0.48 | TEAD2 | 0.40 |
| SNORD85 | 2.38 | IFITM2 | 0.48 | CKMT1A | 0.40 |
| GEM | 2.38 | MORC2-AS1 | 0.48 | SNORD6 | 0.40 |
| SNORD96A | 2.38 | CCDC78 | 0.48 | ADORA1 | 0.40 |
| SNAI2 | 2.36 | LOC401052 | 0.48 | CRLF1 | 0.40 |
| MIR1244-3 | 2.34 | ACBD4 | 0.48 | RNF208 | 0.40 |
| HIST1H2BI | 2.34 | CA5BP1 | 0.48 | LOC100506123 | 0.40 |
| MINOS1-NBL1 | 2.32 | TMEM44 | 0.48 | UCN | 0.39 |
| SNORD56 | 2.32 | BIK | 0.48 | OGDHL | 0.39 |
| LOC102467081 | 2.31 | CERS4 | 0.48 | TMEM86B | 0.39 |
| NEK7 | 2.30 | CGREF1 | 0.48 | SCXB | 0.39 |
| CYB5R4 | 2.23 | SERPINA1 | 0.48 | SLC29A4 | 0.39 |
| MPP4 | 2.22 | PSD | 0.48 | CNFN | 0.39 |
| AMIGO2 | 2.21 | IFITM1 | 0.47 | PCDHGC3 | 0.39 |
| RNF6 | 2.21 | MMEL1 | 0.47 | WFDC2 | 0.39 |
| THBS1 | 2.21 | CDK3 | 0.47 | FAM231D | 0.38 |
| GCLM | 2.20 | SPON2 | 0.47 | YPEL1 | 0.38 |
| VRK2 | 2.20 | PALM | 0.47 | COL1A1 | 0.38 |
| S100P | 2.20 | C20orf96 | 0.47 | LOC644936 | 0.38 |
| TFPI2 | 2.20 | NPTXR | 0.47 | TP53I11 | 0.38 |
| GUSBP4 | 2.19 | ITGAX | 0.47 | UPRT | 0.38 |
| RNVU1-19 | 2.17 | GSTM2 | 0.47 | KRT81 | 0.38 |
| ETNK1 | 2.16 | CAPS | 0.47 | NRTN | 0.37 |
| SRFBP1 | 2.16 | ECHDC2 | 0.47 | BCAM | 0.37 |
| SGPP1 | 2.16 | FANK1 | 0.47 | PRR22 | 0.37 |
| FRMD6-AS1 | 2.15 | LINC00265 | 0.47 | SULT1A4 | 0.37 |
| PTX3 | 2.15 | MYL9 | 0.47 | PTPRCAP | 0.36 |
| SNORD20 | 2.15 | EMID1 | 0.47 | SYTL1 | 0.36 |
| TMEM170A | 2.14 | LRRC26 | 0.47 | BSCL2 | 0.36 |
| TAF13 | 2.14 | VAX2 | 0.47 | HOXC-AS3 | 0.35 |
| SMIM15 | 2.14 | NLGN2 | 0.47 | CD14 | 0.35 |
| SNORD23 | 2.13 | MAGED4B | 0.47 | SYTL4 | 0.35 |
| MAP4K3 | 2.13 | PPDPF | 0.47 | C10orf10 | 0.35 |
| KLF10 | 2.12 | RHOV | 0.47 | PLA2G4B | 0.33 |
| PRKAR2A | 2.12 | PERM1 | 0.47 | CAPN12 | 0.33 |
| DNAJB4 | 2.11 | MEF2B | 0.47 | ACTA2 | 0.32 |
| MCTS2P | 2.11 | HOXB5 | 0.47 | CTXN1 | 0.32 |
| SNORA52 | 2.10 | KRT7 | 0.47 | EIF4EBP3 | 0.32 |
| CD2AP | 2.10 | CEMP1 | 0.46 | UPK1A-AS1 | 0.31 |
| HIST1H1E | 2.10 | MXRA8 | 0.46 | CHRNE | 0.31 |
| NIPSNAP3A | 2.10 | LOC729737 | 0.46 | TIAF1 | 0.30 |
| PSAT1 | 2.10 | KRT86 | 0.46 | SNORD66 | 0.30 |
| SNORA45B | 2.09 | NES | 0.46 | EIF3CL | 0.29 |
| MIR320A | 2.09 | SPEG | 0.46 | SNORD102 | 0.29 |
| CXCL8 | 2.09 | SNORD46 | 0.46 | WNT6 | 0.28 |
| GNAI1 | 2.08 | ADAMTS13 | 0.46 | GP1BB | 0.28 |
| LRRC40 | 2.08 | TCF7L1 | 0.46 | GGT5 | 0.25 |
| KLF15 | 2.08 | DMKN | 0.46 | MIR4516 | 0.21 |
| LACTB2 | 2.08 | ZCCHC24 | 0.46 | SNORA84 | 0.21 |
| HIF1A | 2.08 | CDH24 | 0.46 | TAF9B | 0.03 |
| PKN2 | 2.07 | DBP | 0.46 |  |  |

**Table S2** Primer sequences for plasmid construction.

| **Primer Names** | **Sequences (5’→3’)** |
| --- | --- |
| pri-miR-1246-F | ATTCTAGAGCTAGCGAATTCGGAAACCACTGCTTTCT |
| pri-miR-1246-R | TCCTTCGCGGCCGCGGATCCGTCATAGGTACCATGCC |
| miR-1246-WT-F | ATTCTAGAGCTAGCGAATTCTGTATCCTTGAATGGATTTTTGGAGCAGGAGTGGACACCTGACCC |
| miR-1246-WT-R | TCCTTCGCGGCCGCGGATCCATTGCTAGCCTATGGATTGATTTCCTTTGGGTCAGGTGTCCACTCC |
| miR-1246-Del-F | ATTCTAGAGCTAGCGAATTCTGTATCCTTGATTTGGAGCAGGAGTGGACACCTGACCC |
| miR-1246-Del-R | TCCTTCGCGGCCGCGGATCCATTGCTAGCCTATGGATTGATTTCCTTTGGGTCAGGTGTCCACTCC |
| miR-1246-Mut-F | ATTCTAGAGCTAGCGAATTCTGTATCCTTGACGTACGGTTTGGAGCAGGAGTGGACACCTGACCC |
| miR-1246-Mut-R | TCCTTCGCGGCCGCGGATCCATTGCTAGCCTATGGATTGATTTCCTTTGGGTCAGGTGTCCACTCC |
| miR-1246-locus-WT-F | GGTAAAATCGATAAGGATCCTACAGCCATACATTAGC |
| miR-1246-loucs-WT-R | CAAGGGCATCGGTCGACTAGCTGGTACTATGATCC |
| miR-1246-locus-Mut-F1 | GGTAAAATCGATAAGGATCCTACAGCCATACATTAGC |
| miR-1246-loucs-Mut-R1 | CCGTACGTCAAGGATACAAATCTGAC |
| miR-1246-locus-Mut-F2 | TTGTATCCTTGACGTACGGTTTGGAGCAGGAGTGGA |
| miR-1246-loucs-Mut-R2 | CAAGGGCATCGGTCGACTAGCTGGTACTATGATCC |
| MMP1-locus-WT-F | GGTAAAATCGATAAGGATCCCAAATGAAAGGAAGCCACT |
| MMP1-loucs-WT-R | CAAGGGCATCGGTCGACTTCTCCTACTTCAGCCTCCT |
| MMP1-locus-Mut-F1 | GGTAAAATCGATAAGGATCCCAAATGAAAGGAAGCCACT |
| MMP1-loucs-Mut-R1 | CCGTACGCATTTTGTCATAGAAAAAGACAAAAAT |
| MMP1-locus-Mut-F2 | CTATGACAAAATGCGTACGGAGAAGAACTGACTTGCCCAGAGTCAC |
| MMP1-loucs-Mut-R2 | CAAGGGCATCGGTCGACTTCTCCTACTTCAGCCTCCT |

**Table S3** Primer sequences for RT-qPCR.

| **Primer Names** | **Sequences (5’→3’)** |
| --- | --- |
| U6-F | CTCGCTTCGGCAGCACA |
| U6-R | AACGCTTCACGAATTTGCGT |
| miR-1246-F | CGTCAGCTGTCCGAGTAGAGGAATGGATTTTTG |
| miR-1246-R | TGTCAGGCAACCGTATTCACCCCTGCTC |
| pre-miR-1246-F | GGTCAGATTTGTATCCTTGAATGG |
| pre-miR-1246-R | GGTTGATTGCTAGCCTATGG |
| miR-17-F | CGTCAGCTGTCCGAGTAGAGGCAAAGTGCTTAC |
| miR-17-R | TGTCAGGCAACCGTATTCACCCTACCTG |
| miR-20a-F | CGTCAGCTGTCCGAGTAGAGGTAAAGTGCTTAT |
| miR-20a-R | TGTCAGGCAACCGTATTCACCCTACCTG |
| miR-19a-F | CGTCAGCTGTCCGAGTAGAGGTGTGCAAATCT |
| miR-19a-R | TGTCAGGCAACCGTATTCACCTCAGTT |
| miR-19b-F | CGTCAGCTGTCCGAGTAGAGGTGTGCAAATCC |
| miR-19b-R | TGTCAGGCAACCGTATTCACCTCAGTT |
| miR-16a-F | CGTCAGCTGTCCGAGTAGAGGTAGCAGCACGTAAAT |
| miR-16a-R | TGTCAGGCAACCGTATTCACCCGCCAAT |
| GAPDH-F | CTGGGCTACACTGAGCACC |
| GAPDH-R | AAGTGGTCGTTGAGGGCAATG |
| MMP1-F | ACTACGATTCGGGGAGAAGTG |
| MMP1-R | TTCCGGGTAGAAGGGATTTG |
| THBS1-F | TCCGCCGATTCCAGATGATTCC |
| THBS1-R | ACAGCGAGTCCAGGATCACAGT |
| PTHLH-F | ACGGCGACGATTCTTCCTTCAC |
| PTHLH-R | TGTTGGGAGAGGGCTTGGAGTT |
| CYP1B1-F | AAGGACCTGACCAGCAGAGTGA |
| CYP1B1-R | TGGTGAGCCAGGATGGAGATGA |
| S100P-F | AGCACGCAGACCCTGACCAA |
| S100P-R | TGAAGTCCACCTGGGCATCTCC |
| MTX2-F | TTGGATGGGGAAAGAAGACTC |
| MTX2-R | AAGAAATACGGTTGTGTTCCCA |
| HOXD1-F | ACCTACCCCAAGTCCGTCTCT |
| HOXD1-R | CAGTTCTGTCAGTTGCTTGGTG |
| HOXD3-F | GCCCAATGCTTCTAGCTCCT |
| HOXD3-R | TCTTGTCCTCGCAGCTCTCT |
| HNRNPA3-F | GTGGTGGACCAGGATATGGA |
| HNRNPA3-R | ATTATAGTTCCCACCACCACCA |
| NFE2L2-F | TCCAAAATGTGTAAGACGGGA |
| NFE2L2-R | TCCAAAGTATGTCAATCAAATCCA |
| AGO2-F | AGGCGTGAACAACATCCTG |
| AGO2-R | AGAAAGATGACGGGCTGCT |
| BCAM-F | GAGGTGCGCTTGTCTGTACC |
| BCAM-R | GCATATAATGGTCGTGGGTTCC |
| IGFBP5-F | ACCTGAGATGAGACAGGAGTC |
| IGFBP5-R | GTAGAATCCTTTGCGGTCACAA |
| PDPN-F | GTGTAACAGGCATTCGCATCG |
| PDPN-R | TGTGGCGCTTGGACTTTGT |

**Table S4** Primer sequences for ChIP-qPCR.

| **Primer Names** | **Sequences (5’→3’)** |
| --- | --- |
| miR-1246 locus-F | CATTTGGAGGCGGTCAGATT |
| miR-1246 locus-R | AGGTTGATTGCTAGCCTATGG |
| MMP1 locus-F | CCCTAGTCCAATGCCTTC |
| MMP1 locus-R | GGGAGAGTATTCCAAGGC |

**Table S5** Oligonucleotides for EMSA assay.

| **Oligonucleotide Names** | **Sequence (5’ → 3’)** |
| --- | --- |
| miR-1246 ssRNA | AAUGGAUUUUUGGAGCAGG |
| 5’Cy5-miR-1246 ssRNA | (Cy5) AAUGGAUUUUUGGAGCAGG |
| Anti-miR-1246-ssDNA1 | CCTGCTCCAAAAATCCATT |
| Anti-miR-1246-ssDNA2 | ATGACGCTGCCGAATTCTACCGCCCTGCTCCAAAAATCCATTTTTAGGTTCACCTCGTCCCTGGC |
| 5’Cy5-anti-miR-1246-ssDNA3 | (Cy5) CCTGCTCCAAAAATCCATT |
| ssDNA4 | GCCAGGGACGAGGTGAACCTGGCCCGTTCGGGCGTAGTTCATTATGTAGAATTCGGCAGCGTCAT |
